# Novel VEGFR2 inhibitors with thiazoloquinoxaline scaffold targeting hepatocellular carcinoma with lower cardiotoxic impact

**DOI:** 10.1038/s41598-023-40832-z

**Published:** 2023-08-25

**Authors:** Reham M. M. El-Hazek, Nashwa H. Zaher, Mostafa G. M. El-Gazzar, Noha A. Fadel, Walaa A. El-Sabbagh

**Affiliations:** Drug Radiation Research Department, National Center for Radiation Research and Technology (NCRRT), Egyptian Atomic Energy Authority (EAEA), Cairo, 11787 Egypt

**Keywords:** Chemical biology, Drug discovery

## Abstract

Hepatocellular carcinoma (HCC) is a fatal tumor which is usually diagnosed at advanced stage. Molecular targeted drugs were used recently to treat HCC, however, due to serious side effects, mainly cardiotoxicity and emergence of resistance, there is demanding to explore new chemotherapeutics. 10 novel thiazoloquinoxaline derivatives coupled with different sulfonamide moieties **4(a–j)** were designed and synthesized fulfilling pharmacophoric features of VEGFR-2 inhibition. Structures of all new compounds were verified via spectral and microanalytical data. After carrying in-vitro VEGFR-2 assay for compounds **4(a–j)**; sulfapyridine and sulfamethoxazole derivatives **4d and 4f** showed potential inhibitory effect [61.04 and 83.35 nM], respectively, comparable to standard sorafenib [51.41 nM]. Both were then further evaluated for their cytocidal activity against HepG2 cell-line and against myocardium cells using H9C2 cell-line. As a result, only sulfapyridine derivative **4d** exhibited a significant inhibition of HepG2 cells viability [IC_50_ = 4.31 μM]. Furthermore, it showed relatively lower cytotoxic impact against normal H9C2 myocardium cells [IC_50_, 33.47 μM] compared to that of sorafenib [IC_50_, 98.07 μM]. In-vivo study was carried out to determine myocardium safety of compound **4d** on irradiated mice (8 Gy). In-vivo results of sulfapyridine derivative **4d** showed normal cardiac enzyme function (CK) and serum catalase activity with significant reductions in LDH, cardiac TNF-α and caspase-9 levels, alongside with its efficacy in suppressing the expression of hepatic VEGF. In conclusion, sulfapyridine derivative **4d** could be considered a promising candidate as VEGFR-2 inhibitor with less myocardium side effect.

## Introduction

Hepatocellular carcinoma (HCC), establishes more than 90% of liver’s primary tumor. Recently, HCC constitutes the 5th most common cancer globally. HCC is the 2nd leading cause of cancer-specific mortality in men, after lung cancer and it is usually diagnosed at advanced stage^1^. Chemotherapeutics, either systemic or local, along with surgical and/or radiological intervention is a tactic used to increase HCC patients’ survival rate^2,3^. However, and due to toxic side effects on many organs, the clinical use of traditional chemotherapeutic drugs as 5-fluorouracil, doxorubicin and cisplatin, are significantly being restricted as they owe poor specificity^4^.

Recently, molecular targeted drugs show higher specificity on tumor tissues with better therapeutic effects against HCC^5,6^. Sorafenib, a multi kinase inhibitor, was approved from FDA as a first-line molecular targeted drug for the treatment of advanced HCC since 2007^7,8^. Sorafenib blocked tyrosine kinases receptors; Platelet Derived Growth Factor receptor (PDGFR-β), and Vascular Endothelial Growth Factor Receptor (VEGFR) in endothelial cells, which further inactivate Mitogen Activated Protein Kinase/ Extracellular signal-regulated Kinases (MAPK/ERK) pathways involved in genes transcription that promotes angiogenesis, leading finally to tumor shrinkage, it also inhibits translations of pro-survivors Mcl-1 and Bcl-2, inducing apoptosis in various cancer cells types^9–11^. In spite of sorafenib potency in HCC treatment, primary or acquired resistance develops in many patients^12^. It exerts some disadvantageous side effects due to normal tissues’ non-specific uptake, as well as its narrow therapeutic index which limit its future clinical application^13^. Moreover, FDA approved sorafenib derivatives were explored hoping to overcome all the above-mentioned challenges, but there are still obstacles and encounters to overcome, such as the moderate clinical efficacy, mechanism-related toxicities and the incidence of clinical resistance.

More importantly, the ability of sorafenib to suppress more than 15 types of tyrosine kinases could negatively induce cardiovascular complications due to enzymatic and mitochondrial functions suppression^14–16^. The primary contributing factor to these compounds' undesirable cardiovascular output is the suppression of VEGFR expressed on endothelial and smooth muscle vascular cells, as they exhibited anti-proliferative, anti-angiogenic, and apoptotic manner by acting on the MAPK/ERK pathway and producing reactive oxygen species (ROS). This could further lead to unfavorable clinical outcomes like tissue injury, vascular dysfunction, hypertension, arrhythmia, myocarditis and even myocardial infarction which could limit the treatment protocol of cancer patient and even threatened their lives^17–20^. These complications could be diagnosed by measuring of creatine kinase (CK), an early marker of cardiovascular diseases, which is responsible for cardiac hemodynamics via upregulation of myocardium contractility, vascular resistance and cardiac output^21^. Abundance release of serum lactate dehydrogenase enzyme (LDH), which is mostly found in heart and skeletal muscles tissues, is also associated with myocardium injury^22^.

The cardiovascular risks associated with targeted therapies are not the only risk facing cancer patients, as the inclusion of radiotherapy in the treatment protocol of HCC together with TKIs targeted therapies is well established for better therapeutic outcomes^23,24^. Exposure to radiation therapy is considered another risk for vascular and myocardium tissue damage with high incidence of cardiomyopathy, valvular disease, atherosclerosis and even myocardial infarction^25,26^. Radiation induced endothelial cells dysfunction due to oxidative damage of DNA and production of pro-inflammatory mediators such as TNF-α, IL-1, IL-6, IL-8. Also, dysregulations of lipid metabolism and changes in calcium homeostasis are frequently recognized with radiotherapy^27^. Moreover, radiation induced apoptosis to cardiomyocytes and cardio fibroblasts in a dose dependent matter, while apoptosis of cardiac endothelial cells occurred only after exposure to moderate to high doses of radiation^28,29^.

Exploring novel HCC candidates with high selectivity is often desperately required. Quinoxaline has been proven to be a key moiety in drug design and exploration. It was reported to exert wide range of biological activities^30^. Owing to versatility of derivatives and chemical easiness, quinoxalines are considered promising attractive targets for selective anticancer development. Recently Khandan and co-workers replaced sorafenib’s *N-methylpicolinamide* group with quinoxalinedione for synthesis of sorafenib derivatives with potential cytotoxic effec ^31^.

Sulfonamide hybrids were reported for their potential anticancer efficacy with minimal toxicities^32^. Sayed and co-workers coupled sulfonamide with hydrazone for anticancer activity via VEGFR-2 inhibition^33^. A novel series of diazepam bearing sulfonamide moieties were reported as VEGFR-2 inhibitors^34^.

Seeking for improving lives quality in hepatic cancer patients, this study aims at exploring novel thiazoloquinoxaline derivatives endowed with sulfonamide moieties based upon the main pharmacophoric features for inhibition of VEGFR-2, with less adverse actions on cardiomyocytes.

### Rational and design

Molecular targeted therapies which inhibit tyrosine kinases were a breakthrough in treatment of HCC ^35^. FDA approved many drugs downregulating VEGFRs and act as angiogenesis inhibitors. Pazopanib, was developed as sorafenib derivative in 2011 for treating HCC and it showed promising effectiveness and safety profile in its clinical trials before its approval in 2012^36,37^. After then, Santoti and co-workers reported gastrointestinal toxicities for pazopanib^38^. Other toxicities of pazopanib were then reported as depigmentation phenomena, proteinuria, hepatotoxicity, hypothyroidism, as well as, hypertension, thrombosis and cardiac dysfunction^39^. Despite the reported progress in HCC treatment modalities, there is always an urgent need for HCC drug development with new scaffolds in order to obtain new candidates of better therapeutic index and to combat the continuous emergence of cancer cells’ acquired resistance^40^**.**

Quinoxalines are promising scaffolds for exploring new VEGFR-2 inhibitors and selective anticancer drugs ^41^. Sulfonyl hybrids were reported for anticancer activity through VEGFR-2 inhibition with minimal toxicities^32,42^**.**

**Figure 1:**
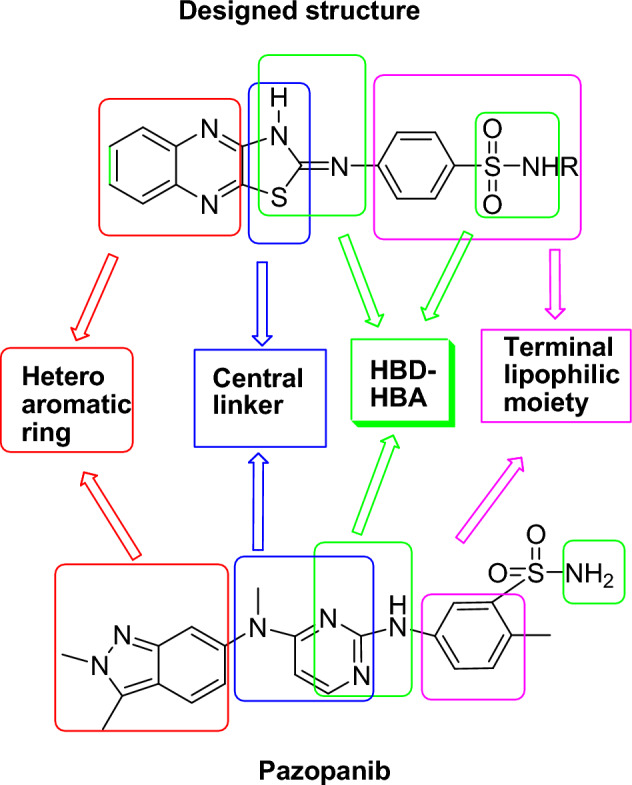
Main pharmacophoric features for VEGFR-2 inhibition using pazopanib as an example.

From all the above mentioned and in continuation to our efforts in exploring new candidates against HCC and as VEGFR-2 inhibitors^43^, herein we designed and synthesized a novel series of thiazoloquinoxaline derivatives coupled with sulfonamide moieties following the four pharmacophoric features required for occupying VEGFR-2 active binding site^44^. Pazopanib was taken as an example along with some bioisosteric modifications (Fig. 1). 1- Quinoxaline ring in our designed structures acts as the heteroaromatic moiety for occupying the hinge region, expecting to offer more binding interactions than pyridine ring of sorafenib and as a bioisoster to the indazole ring of pazopanib. This comes in accordance with Yu and co-workers^45^**,** who reported that substitution of sorafenib pyridine ring and urea contributed in more kinase targeting effect 2- Thiazole ring is the central aromatic linker in our designed compounds, to occupy the gate keeper region, where Chen and co-workers^46^ reported that 1,3-substituted ring display superior activity in VEGFR-2 inhibition**,** in addition to offering of more regioselectivity and stability. 3-Thiazole ring along with central imine, supposed as HBD-HBA pharmacophore, aiming to form H- bonds for inhibitory activity against VEGFR-2, thus resembling the amino pyrimidine ring of pazopanib. 4- The terminal sulfonyl phenyl ring is nominated to occupy the allosteric lipophilic pocket.

## Results and discussion

### Chemistry

Different sulfonamide substituents were used in our study as bioisostere to the sulfonyl terminal of pazopanib in order to investigate their interactions and effect on orientation within active binding site of VEGFR-2, which consequently may result in more residue interactions and probably more stabilization of newly synthesized compounds.

**Scheme 1:**
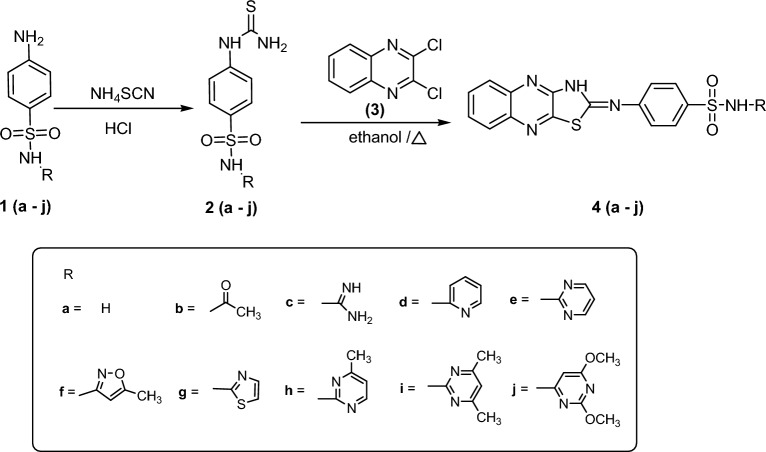
Synthetic route of compounds **4 (a–j)**.

The novel target compounds were synthesized according to the pathways described in Scheme 1. A series of different substituted sulfonamides **1 (a–j)** were reacted with ammoniumthiocyanate to yield thioureidobenzenesulfonamide derivatives **2 (a–j)**^47,48^, respectively. To couple quinoxaline moiety with sulfonamide moieties, compounds **2 (a– j)** were further reacted with 2,3-dichloroquinoxaline **(3)** to give the novel target *N-(substituted)*-4-(thiazolo[4,5-b]quinoxalin-*2(3H)*-ylideneamino)-benzenesulfonamide derivatives **4 (a–j)** respectively, in considerable yields.

All the new compounds obtained were characterized and confirmed by spectral and microanalytical data.

Structure of the novel targeted *N-(substituted)*-4-(thiazolo[4,5-b]quinoxalin-*2(3H)*-ylideneamino)benzenesulfonamide derivatives **4 (a–j)** were characterized by the disappearance of the NH_2_ and C=S bands of the thiourea in all their respective IR spectra and also the disappearance of the NH_2_ signals in their ^1^H NMR spectra exchangeable with D_2_O. Furthermore, ^13^C NMR spectra of compounds **4 (a–j)** displayed characteristic signals of N=C–S at range 135.5–137.4 ppm. Signals of C=N also appeared at range 160.1–162.2 ppm. Signals of N=C–NH were shown at range 162.1–164.1 ppm. All data obtained confirmed incorporation of NH_2_ and C=S of thiourea intermediates **2 (a–j)** in cyclization upon reaction with 2,3-dichloroquinoxaline **(3)** to form the novel targeted thiazoloquinoxaline derivatives **4 (a–j)**. While by more observation of their spectra, they showed the appearance of some significant signals of the sulfonamide series which had been used. For example, ^1^H-NMR spectrum of compound **(4a)** displayed singlet signal at 2.43 ppm, exchangeable with D_2_O, for NH_2_. ^1^H-NMR spectrum of compound **(4b)** showed the presence of singlet signal at 2.10 ppm attributed to CH_3_ group, while it appeared as the most shielded C at 21.2 ppm in its ^13^C NMR spectrum. ^1^H-NMR spectrum of compound **(4c)** exhibited singlet signals at 8.18, 8.34 ppm exchangeable with D_2_O for NH, NH_2_, respectively. while its ^13^C- NMR spectrum showed a downfield signal at 158.4 ppm attributed to C guanidine (NH=C–NH_2_) formed. ^1^H-NMR spectrum of compound **(4d)** displayed a triplet and two doublet signals at 6.71, 6.79, 7.85 ppm respectively for the CHs-pyridine ring. While ^13^C NMR spectrum of compound **(4d)** showed signals 136.4–148.4 pm for CHs pyridine ring and a signal at 152.7 ppm attributed (NH-C-pyridine). ^1^H-NMR spectrum of compound **(4e)** revealed triplet and doublet signals at 6.84 and 8.64 ppm, respectively ascribed for aromatic hydrogens of pyrimidine ring. ^13^C-NMR spectrum of compound **(4e)** displayed signals at 117.5, 157.1 ppm for CH-pyrimidine while the C-pyrimidine ring appeared at 168.5 ppm. ^1^H-NMR spectrum of compound **(4f.)** showed singlet signal at 6.12 ppm attributed to CH of isoxazole ring, while C of isoxazole CH appeared at 95.4 ppm in ^13^C-NMR spectrum. ^1^H-NMR spectrum of compound **(4 g)** displayed two doublet signals at 6.28, 6.86 ppm for 2 CH thiazole ring introduced. ^13^C-NMR spectrum of compound **(4g)** showed signals at 112.1, 136.3 ppm ascribed for 2CH thiazole ring. ^1^H-NMR spectrum of compound **(4h)** exhibited an upfield signal at 2.43 ppm of the methyl group. In addition to two doublet signals appeared at 6.83 and 7.97 ppm for the hydrogens of 2CH-pyrimidine ring while their Cs were displayed at 156.3, 169.2 in its respective ^13^C-NMR spectrum. ^1^H-NMR spectra of compounds **(4i) and (4j)** revealed upfield singlet signals at 2.33 ppm (2 CH_3_) and 3.75, 3.87 ppm (2 OCH_3_) groups, of sulfamethazine and sulfadimethoxine, respectively, while their Cs appeared at 24.0 ppm and 54.4 ppm, respectively at their corresponding ^13^C-NMR spectra.

In addition, the mass spectra and microanalytical data of compounds **4 (a–j)** were in agreement with their postulated structures.

### Biological evaluation

#### In-vitro assay

VEGF receptors are highly expressed in many tumor types and their prolonged presence indorsed vascular network, promoting tumor growth and metastases. VEGFR2 is the type II of tyrosine kinase transmembrane receptor and it is the principal angiogenic signaling mediator in tumors^49^. First, we carried in-vitro assay to compare VEGFR-2 suppression activities among the FDA approved VEGFR-2 inhibitor (Sorafenib) and our newly synthesized compounds **4 (a–j)**. Results showed that IC_50_ of sulfapyridine derivative **4d** and sulfamethoxazole derivative **4f**, was as low as the reference drug, sorafenib (Table. 1).

**Table 1:** In-vitro VEGFR-2 inhibition assay.

Compound	VEGFR-2 IC_50_ (nM)
**4a**	169.40 ± 7.40
**4b**	132.05 ± 5.70
**4c**	253.60 ± 11.00
**4d**	61.04 ± 2.60
**4e**	112.17 ± 4.80
**4f**	83.35 ± 3.70
**4g**	99.76 ± 4.30
**4h**	217.36 ± 9.40
**4i**	287.50 ± 13.00
**4j**	368.16 ± 16.00
**Sorafenib**	51.41 ± 2.30

IC50 values are the mean ± S.D (standard deviations) of three experiments.

Accordingly, the two promising compounds **4d** and **4f** along with sorafenib had been further investigated for their cytotoxic anticancer activity against human HepG2 cell line. Results showed that sorafenib as well as sulfapyridine derivative **4d** significantly decreased cell viabilities and achieved IC_50_ at concentrations of 2.97 μM and 4.31 μM, respectively (Table 2). The cytotoxic activity of sorafenib referred mainly to its ability to suppress the VEGFR-2, exerting its anti-angiogenic and apoptotic effects ^50,51^. Moreover, the suppressing potency of compound **4d** on HepG2 cells proliferation (4.31 nM) was in line with earlier research, demonstrated the cytotoxic effects of sulfonamide derivatives, particularly sulfapyridine ^52,53^.

**Table 2:** Effect of sorafenib, compounds 4d and 4f on HepG2 cells viability.

Conc	HepG2 viability %					IC_50_ (μM)
	0.01 μM	0.1 μM	1 μM	10 μM	100 μM	
Sorafenib	99.58 ± 0.94	98.22 ± 3.19	87.09 ± 2.02	11.32 ± 0.31	4.05 ± 0.1	2.97 μM
4d	98.55 ± 0.47	98.32 ± 0.97	98.09 ± 1.8	6.62 ± 0.5	3.81 ± 0.24	4.31 μM
4f	97.57 ± 1.26	97.09 ± 1.06	96.6 ± 1.2	95.48 ± 3.0	93.95 ± 2.03	> 100 μM

Data are expressed as mean ± S.D (standard deviations) of three experiments.

**Figure 2:**
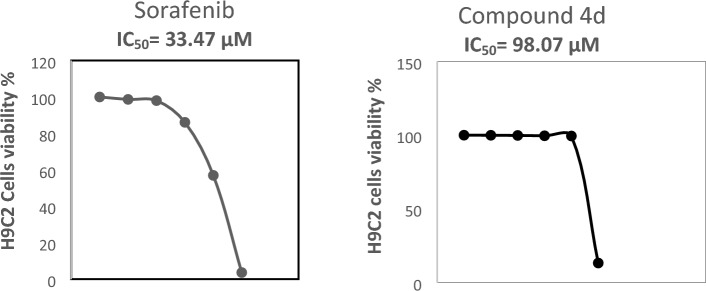
Effect of sorafenib and compound 4d [0.01, 0.1, 1, 10 and 100 μM] on H9C2 cells viability and IC_50_.

Based on the previous results, a further comparable screening between compound **4d** and sorafenib on cardiomyocytes viability was carried out on H9C2 cell culture; results of compound **4d** showed 2.93 times less cytotoxic than sorafenib **(**Fig. 2**)**. Sorafenib interferes with the electron transport system by creating ROS, which led to ATP depletion, consequently causing proteins damage in the mitochondrial membrane, leading to an irreversible loss of membrane potential. This was reported to be the primary cause of sorafenib cardiotoxicity ^16^. Moreover, cardiac toxicity of sorafenib could be attributed also to depletion of taurine which plays a major cellular protective role as an anti-oxidant, stabilizing proteins factor and neuro-modulating agent and its lack promoted surging of NF-Kβ, P53 and P38 leading to systolic and diastolic abnormalities and even myocardial infarction ^54,55^.

#### Acute oral toxicity test

The *in-vivo* acute toxicity signs associated with compound **4d** were also assessed. No mortalities, weight loss, or behavioral alterations were seen in mice treated with 200, 500, 800, or 1000 mg/kg of compound 4d up to14 days. Overall, compound 4d displayed a high level of safety up to 1000 mg/kg.

#### In-vivo assay

Cardiovascular dysfunction is a significant risk connected with exposure to γ-radiation, particularly for cancer patients who may also be given tyrosine kinase inhibitors concurrently^23,24^. Radiotherapy could exert many adverse cardiac reactions such as myocardial infarction, hypertension, vascular dysfunction, apoptosis and even necrosis of normal heart tissue^25,56^.

**Figure 3:**
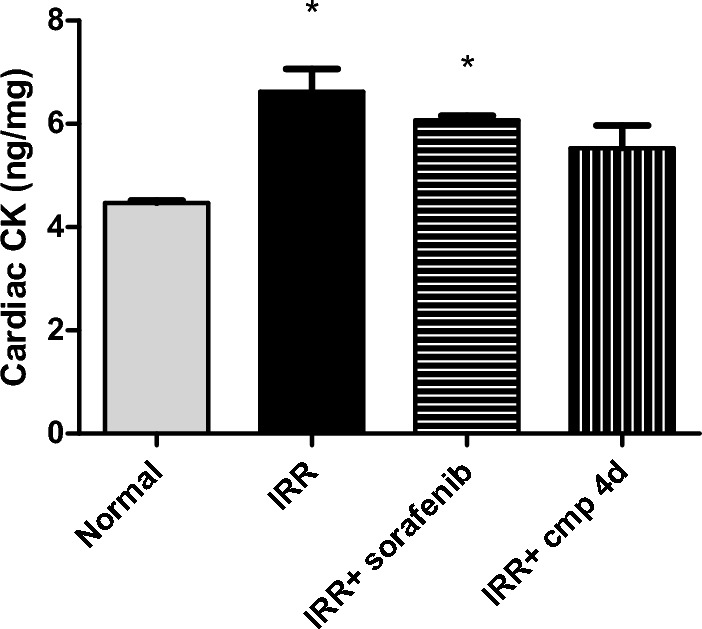
Effect of daily treatment (3 days) of sorafenib or compound 4d [50 mg/ kg, p.o] on heart creatine kinase activity (CK, ng/mg) in 8 Gy irradiated mice. All values are plotted as mean ± S.E. * represented significance from Normal at *P* ≤ 0.05, # represented significance from IRR at *P* ≤ 0.05, s represented significance from IRR + sorafenib at *P* ≤ 0.05.

Owing to this risk, a demand to new targeted therapies with less cardiotoxic effect was the goal of this study. In order to evaluate the cardiac adverse consequences, we screened in the current study the cardiac function of irradiated mice after treatment with either sorafenib or compound **4d**. First, CK enzyme activity was determined as it is one of the earliest diagnostic markers for cardiac dysfunction. Significant increments in CK enzymatic activity by 48.35% and 35.88% in IRR group and IRR + sorafenib group, respectively, had been recorded (Fig. 3). Even though compound **4d** did not significantly lower CK level compared to the IRR non-treated group, it was intriguing to see that CK activity of compound 4d group was still not significantly different from normal values (Fig. 3).

In this investigation, serum levels of LDH and catalase were tested to examine the state of general oxidative stress and tissue damage. LDH is found in most organ tissues and has a functional role in gluconeogenesis and DNA metabolism. Its elevation is indicative of various disorders, including cardiac illnesses, and is a helpful diagnostic marker for tissue damage, as well as energy homeostasis^22,57^. One of the essential antioxidant enzymes is catalase, as it breaks down cellular hydrogen peroxide to create water and oxygen, reducing oxidative stress to a significant degree, and its deficiency may contribute to the development of a variety of disorders^58^.

**Figure 4:**
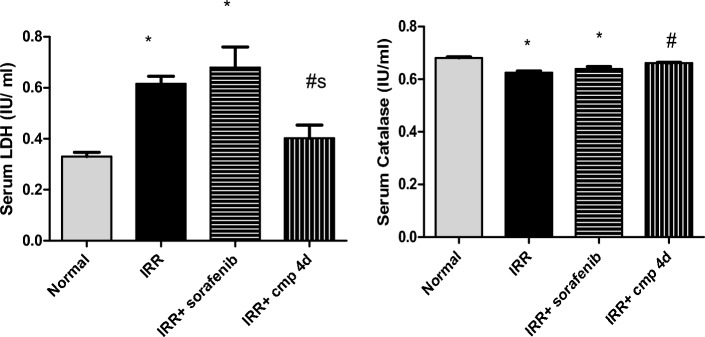
Effect of daily treatment (3 days) of sorafenib or compound 4d [50 mg/ kg, p.o] on serum lactate dehydrogenase (LDH, IU/ml) and Catalase activity (IU/ml) in 8 Gy irradiated mice. All values are plotted as mean ± S.E. * represented significance from Normal at *P* ≤ 0.05, # represented significance from IRR at *P* ≤ 0.05, s represented significance from IRR + sorafenib at *P* ≤ 0.05.

Both IRR and IRR + sorafenib groups experienced substantial increases in serum LDH by 86.66% and 106.06%, respectively, while IRR + compound **4d** group exhibited a non-significant deviation from normal LDH values. Furthermore, a significant decrease in serum LDH of compound 4d treated group by 40.83%, compared to sorafenib treated group, revealed better protection from tissue damage following radiation exposure. In the same line, IRR and IRR + sorafenib groups had shown substantial decreases in serum catalase activities by 8.22% and 6.19%, respectively while the IRR + comp 4d group had normal catalase activity (Fig. 4). However, this might be explained by the antioxidant properties of sulfur contains compounds, which could reduce the production of endogenous ROS in biological tissue^59,60^.

**Figure 5:**
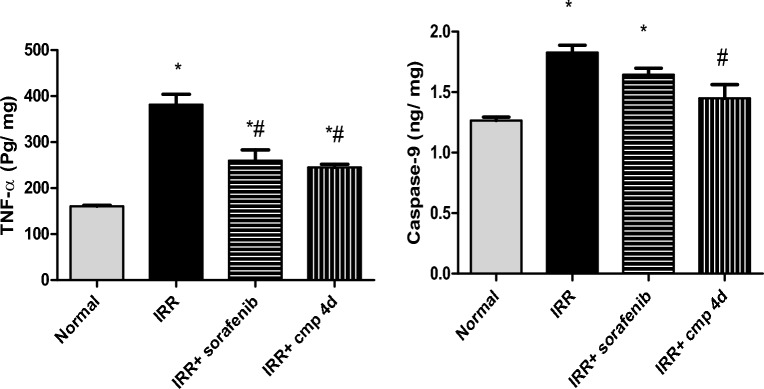
Effect of daily treatment (3 days) of sorafenib or compound 4d [50 mg/ kg, p.o] on heart levels of caspase-9 (ng/mg Tp) and TNF-α (pg/ mg Tp) in 8 Gy irradiated mice. All values are plotted as mean ± S.E. * represented significance from Normal at P ≤ 0.05, # represented significance from IRR at P ≤ 0.05, s represented significance from IRR + sorafenib at P ≤ 0.05.

In the introduced work, γ-irradiation induced a significant increase in cardiac TNF-α by 137.69%, compared to normal values (Fig. 5**)**. Myocardial inflammation can develop shortly after exposure to γ- radiation and can often last for years. However, a number of variables, including adhesion molecules (I-CAM & V-CAM), interleukins, leukotrienes, and prostaglandins, contribute to the pathogenicity of these inflammatory situations ^29^.

It was worth noting that irradiated mice given sorafenib or compound **4d** demonstrated considerable reduction in cardiac TNF-α by 31.87% and 35.75%, respectively, in comparison to the control irradiated group **(**Fig. 5**)**. Another study suggested that sorafenib, as a tyrosine kinase inhibitor, has a role in NF-κB inactivation via inhibiting p-38 phosphorylation, which ultimately results in the suppression of inflammatory cytokines. This attribute could explain the above findings ^61^**.**

Caspase-9 had been tested in this work as a pro-apoptotic marker. Results showed that administration of compound **4d** to irradiated mice significantly decreased Caspase-9 by 20.63%, compared to control irradiated animals **(**Fig. 5**)**. However, cardiac caspase-9 levels were significantly increased in the IRR and IRR + sorafenib groups, by 44.2% and 29.83%, respectively, over normal values **(**Fig. 5**)**. ROS generated from radiation induced activation of Bax which disrupts the permeability of mitochondrial membrane and release cytochrome c into the cytosol, followed by activation of pro-apoptotic caspase-9, initiating caspase-3 cascade leading finally to apoptosis ^29,62,63^. Moreover, the apoptotic effect of sorafenib on cardiomyocytes could be attributed to the affinity of sorafenib to different tyrosine kinase receptors subtypes ^63^. We speculated that the sulfapyridine group incorporated in compound **4d**, has a favorable effect on oxidative state as demonstrated by catalase activity, this could account for the considerable reduction of cardiomyocytes' caspase-9 and, consequently, reduced cardiac apoptotic state. This comes in accordance with Couto and co-workers ^64^ who reported antioxidant effect of sulfapyridine.

**Figure 6:**
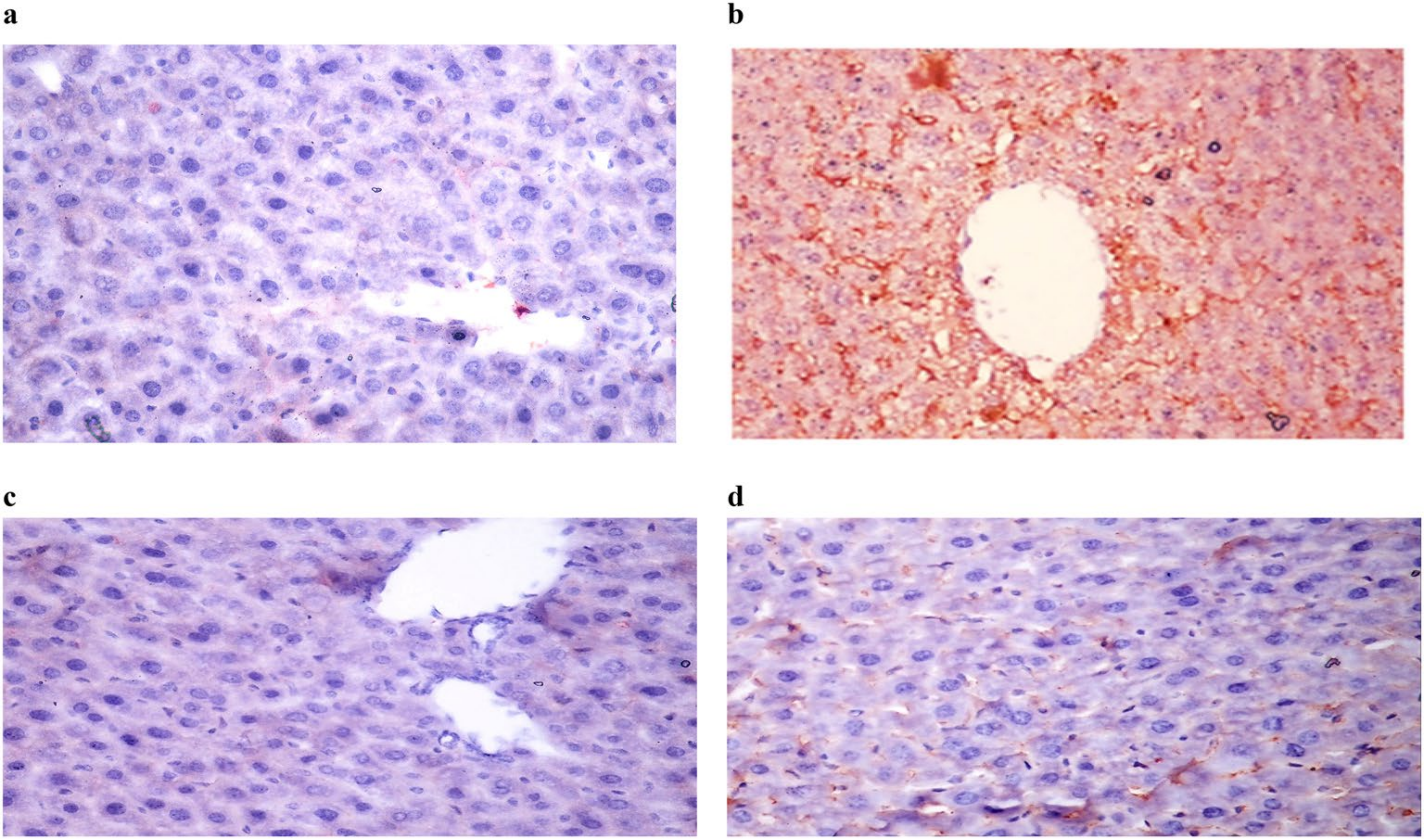
*In-vivo* Immunohistochemistry expression of VEGF in liver tissue of irradiated (8 Gy) mice after daily treatment of sorafenib or compound 4d (50 mg/kg, 3 days, p.o). (a) Normal group, (b) IRR group, (c) IRR + sorafenib group, (d) IRR + compound 4d group (VEGF X400).

The suppression activity of the chosen, promising sulfapyridine derivative **4d** and sorafenib on hepatic VEGF expression in irradiated mice was further estimated and compared. However, it was difficult for antiangiogenic compounds to demonstrate their full effectiveness in presence of γ-radiation, as the later encourages tissue vascularization and perfusion in order to improve oxygenation for further production of ROS needed for DNA damage in tumor cells ^65^. In the current study, the expression of VEGF in liver tissue was evaluated after daily administration with either compound **4d** or sorafenib for 3 days following γ- irradiation (8 Gy). Results obtained showed a strong positive expression of VEGF in irradiated animals, which was promisingly suppressed upon treatment with sorafenib as well as compound **4d** (Fig. 6**)**.

#### Molecular modelling

In silico evaluation using molecular docking was performed in order to confirm and interpret the preliminary VEGFR-2 inhibition results for the most potent newly synthesized compound **4d**. MOE software was used with human VEGFR-2 (PDB: 1ASD). Docking study was performed for the most active compound ; **N-(pyridin-2-yl)-4-(thiazolo[4,5-b]quinoxalin-2(3H)-ylidene-amino) benzenesulfonamide (4d)**, within the active binding site of VEGFR-2 in order to have more perception about the binding mode, taking into consideration main pharmacophoric features required for binding in order to downregulate VEGFR-2 activity as reported^42^.

**Figure 7:**
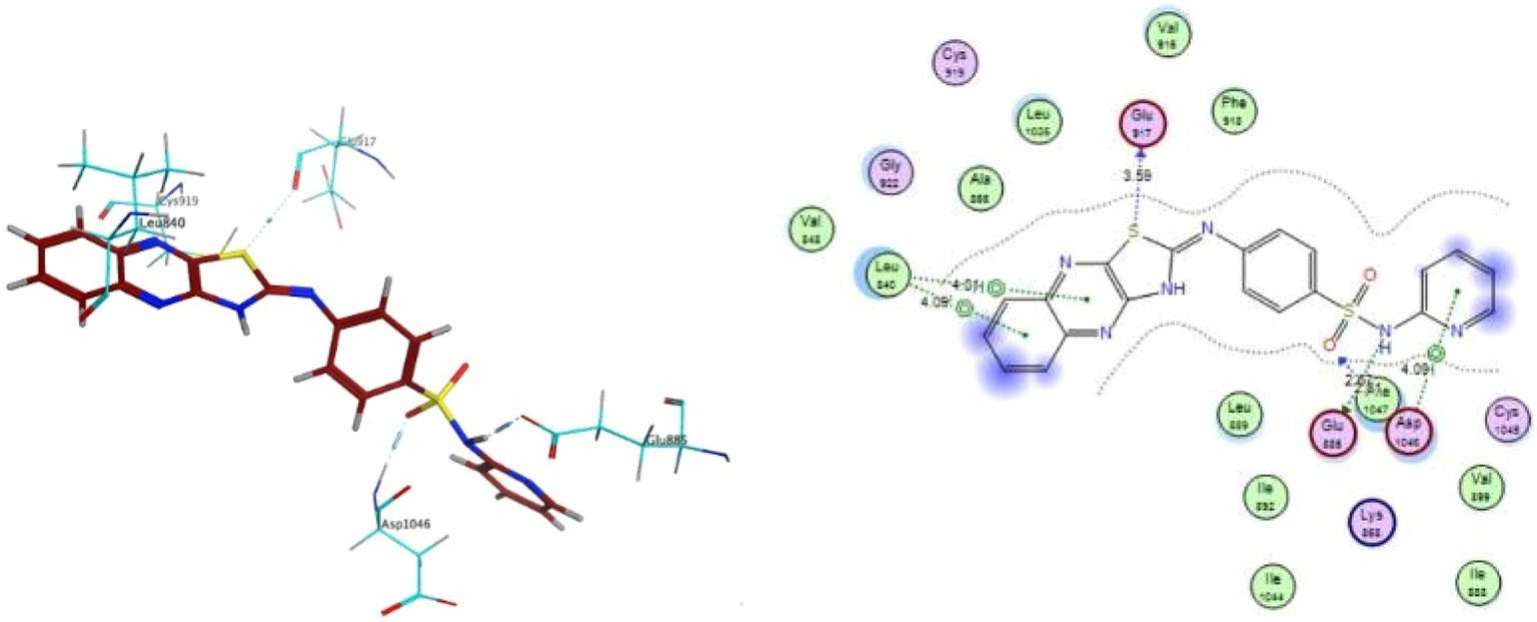
3D and 2D binding interactions of compound **4d** into VEGFR-2 (PDB: 4ASD).

It could be noticed that structure of compound **4d** occupies mainly the catalytic site of VEGFR-2 receptor, which potentially could be reason for its selectivity. It displayed better binding energy at -7.933 compared to -6.953 of pazopanib (supplementary data). It fits through the following binding interactions (Fig. 7): Quinoxaline ring occupies hinge region. Pyrimidine of the quinoxaline occupied the gatekeeper region as central aromatic linker. At the same time, it forms arene binding interaction with leu 840 in DFG domain (HBD-HBA pharmacophore). Sulfur of thiazole ring forms additional main H- bond with Glu 917 in DFG domain, Sulfonamide moiety forms additional 2- H–bonds with Glu 885 and Phe 1047. Phenyl ring of pazopanib forms only one arene interaction with Leu 840, while quinoxaline ring of 4d forms two arene interactions with Leu 840. These additional interactions are missing in pazopanib binding interactions. which could be responsible for the more stability of 4d-VEGFR-complex. Terminal pyridine ring forms the essentiale arene interaction with Asp 1046 and occupies the allosteric lipophilic pocket.

## Conclusion

Based on quinoxaline scaffold and pharmacophore features needed for VEGFR-2 inhibition, herein 10 novel thiazoloquinoxaline derivatives coupled with different sulfonamide moieties 4(a-j) were designed and synthesized. Compounds 4d and 4f. demonstrated strong VEGFR-2 inhibition, and both of them had antiproliferative activity against the HepG2 cell line, indicating in-vitro potential efficacy against HCC. Moreover, compound 4d showed more in-vitro myocardium cytoprotective effect than the commonly used anti-HCC, sorafenib. The therapeutic outcomes of compound 4d and sorafenib were extended for studying in irradiated mice, where both treatments inhibited hepatic VEGF expression. The main advantage of using compound 4d in the current work is the low radiation-cardiotoxic potential, as it diminished the elevated pro-apoptotic, pro-inflammatory and oxidative mediators in myocardium of irradiated mice. Collectively, the current study revealed the efficacy of the newly synthesized N-(pyridin-2-yl)-4-(thiazolo[4,5-b]quinoxalin-2(3H)-ylideneamino) benzene-sulfonamide (4d) as down regulator to VEGFR-2 activity and a cytotoxic agent against HCC. Also, compound 4d has a significant influence on lowering radiation-induced deleterious effects on cardiac tissue, indicating its potential as a cardio-safe VEGFR-2 inhibitor.

## Experimental

### Chemistry

#### General

Uncorrected melting points were recorded using a Stuart melting point device (Stuart Scientific, Redhill, UK) and were transported in open capillary tubes. The infrared (IR) spectra of the substances were captured using an FTIR Shimadzu spectrometer (Shimadzu, Tokyo, Japan). TMS was utilised as an internal Standard and DMSO-d6 as the solvent for recording 1H NMR and 13C NMR spectra with a Bruker (400 MHz for 1H NMR and 100 MHz for 13C NMR) spectrometer. On the HP Model MS-5988, mass spectra were conducted (Hewlett Packard, Palo, Alto, Calofornia, USA). For acquiring the results of the microanalyses, a Carlo Erba 1108 Elemental Analyzer (Heraeus, Hanau, Garmany) was employed. In order to check the completion of the reaction, pre-coated SiO2 gel (HF254, 200 mesh) aluminium plates (Merk, Daemstadt, Germany) were employed as the TLC. CAS number for reagents used are mentioned between brackets following each reagent.

### General procedure for the synthesis of N-(substituted)-4-thioureidobenzenesulfonamide derivatives 2 (a–j)

A mixture of benzenesulfonamide derivatives **1 (63–74-1, 144–80-9, 57–67-0, 144–83-2, 68–35-9, 723–46-6, 72–14-0, 127–79-7, 57–68-1, 122–11-2) (a-j),** respectively (0.01 mol) in diluted HCl 10% (20 ml) was refluxed for 1 h. Ammoniumthiocyanate (1762–95-4) (0.01 mol, 0.76 gm) was then added and the mixture was refluxed for 5 h. The reaction mixture was cooled, poured onto ice water, the precipitated solid was filtered, washed with water, dried and crystallized from ethanol (64–17-5) to give compounds **2(a-j),** respectively.

### 4-Thioureidobenzenesulfonamide (2a)^49^

#### *N-((4-Thioureidophenyl)sulfonyl)* acetamide (2b)

Yield % 75; m.p.; 181–183 °C; IR (KBr,cm^-1^): 3420, 3323, 3275 (NH_2_, 2NH), 3062 (CH arom.), 2915, 2835 (CH aliph.), 1795 (C=O), 1351 (C=S), 1210, 1115 (SO_2_); ^1^H NMR (DMSO-*d*_6_, δ, ppm): 2.06 (s, 3H, CH_3_), 6.46 (d, 2H, Ar–H, *J* = 10.88 Hz), 7.20 (d, 2H, Ar–H, *J* = 8.08 Hz), 8.13 (s, 2H, 2NH, D_2_O exch.), 8.46 (s, 2H, NH_2_, D_2_O exch.). ^13^C NMR (DMSO-*d*_6_, δ, ppm): 21.8 (CH_3_), 123.0 (2), 129.8 (2), 136.8, 140.8, 171.7 (C=O), 181.8 (C=S). MS (m/z): 273(M^+^). Analysis calculated for: C_9_H_11_N_3_O_3_S_2_ (273): C, 39.55; H, 4.06; N, 15.37; found: C, 39.23; H, 4.33; N, 15.58.

#### ***N-Carbamimidoyl*-4-thioureidobenzenesulfonamide (2c)**

Yield % 86; m.p.; 220–222 °C; IR (KBr,cm^-1^): 3395–3145 (3NH, 2NH_2_), 3070 (CH arom.), 1345 (C=S), 1232, 1129 (SO_2_); ^1^H NMR (DMSO-*d*_6_, δ, ppm): 6.08 (d, 2H, Ar–H, *J* = 17.28 Hz), 7.49 (d, 2H, Ar–H, *J* = 7.48 Hz), 8.40 (s, 2H, 2NH, D_2_O exch.), 8.48 (s, H, NH, D_2_O exch.), 8.48 (s, 4H, 2NH_2_, D_2_O exch.) . ^13^C NMR (DMSO-*d*_6_, δ, ppm): 121.2 (2), 129.6 (2), 136.4, 139.9, 159.0 (C=NH), 181.3 (C=S). MS (m/z): 273(M^+^). Analysis calculated for: C_8_H_11_N_5_O_2_S_2_ (273): C, 35.15; H, 4.06; N, 25.62; found: C, 34.86; H, 3.87; N, 25.87.

#### ***N-(Pyridin-2-yl)*-4-thioureidobenzenesulfonamide (2d)**

Yield % 90; m.p.; 180–182 °C; IR (KBr,cm^-1^): 3320, 3284, 3160 (NH_2_, 2NH), 3077 (CH arom.), 1350 (C=S), 1244, 1110 (SO_2_); ^1^H NMR (DMSO-*d*_6_, δ, ppm): 6.35–6.42 (m, 3H, Ar–H), 6.49 (d, H, CH-pyridine, *J* = 3.16 Hz), 7.55–7.68 (m, 3H, Ar–H), 7.86 (d, H, CH-pyridine, *J* = 6.96 Hz), 8.64 (s, 2H, 2NH, D_2_O exch.), 8.66 (s, 2H, NH_2_, D_2_O exch.). ^13^C NMR (DMSO-*d*_6_, δ, ppm): 109.5, 119.6, 123.3 (2), 129.9 (2), 136.4, 139.3, 143.0, 148.4 (CH-pyridine), 152.7 (NH-C-pyridine), 181.8 (C=S). MS (m/z): 308(M^+^). Analysis calculated for: C_12_H_12_N_4_O_2_S_2_ (308): C, 46.74; H, 3.92; N, 18.17; found: C, 46.46; H, 4.25; N, 17.96.

#### ***N-(Pyrimidin-2-yl)*-4-thioureidobenzenesulfonamide (2e)**

Yield % 82; m.p.; > 260 °C; IR (KBr,cm^-1^): 3330, 3270, 3120 (NH_2_, 2NH), 3050 (CH arom.), 1343 (C=S), 1238, 1125 (SO_2_); ^1^H NMR (DMSO-*d*_6_, δ, ppm): 6.35 (d, 2H, Ar–H, *J* = 8.76 Hz), 6,39 (t, H, CH-pyrimidine, *J* = 6.16 Hz), 7.59 (d, 2H, Ar–H, *J* = 5.28 Hz), 8.17 (d, 2H, 2 CH-pyrimidine, *J* = 10.48 Hz), 8.75 (s, 2H, 2NH, D_2_O exch.), 8.89 (s, 2H, NH_2_, D_2_O exch.). ^13^C NMR (DMSO-*d*_6_, δ, ppm): 115.8, 121.7 (2), 129.9 (2), 136.2, 142.1, 156.2 (2), 169.3 (NH–C-pyrimidine), 181.0 (C=S). MS (m/z): 309(M^+^). Analysis calculated for: C_11_H_11_N_5_O_2_S_2_ (309): C, 42.71; H, 3.58; N, 22.64; found: C, 42.39; H, 3.79; N, 22.83.

#### ***N-(5-Methylisoxazol-3-yl)*-4-thioureidobenzenesulfonamide (2f.)**

Yield % 79; m.p.; > 260 °C; IR (KBr,cm^-1^): 3355, 3290, 3263 (NH_2_, 2NH), 3063 (CH arom.), 2978, 2812 (CH aliph.), 1348 (C=S), 1225, 1145 (SO_2_); ^1^H NMR (DMSO-*d*_6_, δ, ppm): 2.41 (s, 3H, CH_3_), 6.11 (s, H, CH-isoxazole), 6.54 (d, 2H, Ar–H, *J* = 4.44 Hz), 7.61 (d, 2H, Ar–H, *J* = 7.28 Hz), 8.30 (s, 2H, 2NH, D_2_O exch.), 8.87 (s, 2H, NH_2_, D_2_O exch.). ^13^C NMR (DMSO-*d*_6_, δ, ppm): 12.8 (CH_3_), 95.3 (CH-isoxazole), 121.0 (2), 130.4 (2), 136.2, 141.4, 150.3 (NH–C-ixoxazole), 169.8 (C–CH_3_), 181.5 (C=S). MS (m/z): 312(M^+^). Analysis calculated for: C_11_H_12_N_4_O_3_S_2_ (312): C, 42.30; H, 3.87; N, 17.94; found: C, 42.11; H, 4.13; N, 18.26.

#### ***N-(Thiazol-2-yl)-*4-thioureidobenzenesulfonamide (2g)**^50^

##### ***N-(4-Methylpyrimidin-2-yl)*-4-thioureidobenzenesulfonamide (2h)**

Yield % 77; m.p.; > 260 °C; IR (KBr,cm^-1^): 3349, 3277, 3125 (NH_2_, 2NH), 3077 (CH arom.), 2923, 2845 (CH aliph.), 1342 (C=S), 1222, 1115 (SO_2_); ^1^H NMR (DMSO-*d*_6_, δ, ppm): 2.46 (s, 3H, CH_3_), 6.34 (d, 2H, Ar–H, *J* = 5.76 Hz), 6.92 (d, H, CH-pyrimidine, *J* = 8.32 Hz), 7.58 (d, 2H, Ar–H,* J* = 4.76 Hz), 8.41 (d, H, N–CH-pyrimidine, *J* = 9.56 Hz), 8.61 (s, 2H, 2NH, D_2_O exch.), 8.89 (s, 2H, NH_2_, D_2_O exch.). ^13^C NMR (DMSO-*d*_6_, δ, ppm): 23.8 (CH_3_), 110.5, 123.5 (2), 129.8 (2), 134.4, 141.0 (CH-pyrimidine), 157.1 (CH-pyrimidine), 166.4 (C–CH_3_), 168.2 (NH–C-pyrimidine), 181.1 (C=S). MS (m/z): 323(M^+^). Analysis calculated for: C_12_H_13_N_5_O_2_S_2_ (323): C, 44.57; H, 4.05; N, 21.66; found: C, 44.24; H, 3.78; N, 21.94.

##### ***N-(4,6-Dimethylpyrimidin-2-yl)*-4-thioureidobenzenesulfonamide (2i)**

Yield % 83; m.p.; 220–222 °C; IR (KBr,cm^-1^): 3388, 3213, 3165 (NH_2_, 2NH), 3059 (CH arom.), 2916, 2855 (CH aliph.), 1349 (C=S), 1208, 1130 (SO_2_); ^1^H NMR (DMSO-*d*_6_, δ, ppm): 2.31 (s, 6H, 2 (CH_3_)), 6.39 (d, 2H, Ar–H, *J* = 6.68 Hz), 6.92 (s, H, CH-pyrimidine), 7.56 (d, 2H, Ar–H, *J* = 9.60 Hz), 8.37 (s, 2H, 2NH, D_2_O exch.), 8.74 (s, 2H, NH_2_, D_2_O exch.). ^13^C NMR (DMSO-*d*_6_, δ, ppm): 24.1 (2) (2CH_3_), 110.4 (CH-pyrimidine), 123.7 (2), 129.9 (2), 135.9, 141.6, 166.5 (2) (2 (C–CH_3_)), 168.9 (NH–C-pyrimidine), 181.3 (C=S). MS (m/z): 337(M^+^). Analysis calculated for: C_13_H_15_N_5_O_2_S_2_ (337): C, 46.27; H, 4.48; N, 20.76; found: C, 45.92; H, 4.74; N, 21.13.

##### ***N-(2,6-Dimethoxypyrimidin-4-yl)*-4-thioureidobenzenesulfonamide (2j)**

Yield % 74; m.p.; 198–200 °C; IR (KBr,cm^-1^): 3340, 3282, 3163 (NH_2_, 2NH), 3086 (CH arom.), 2895, 2810 (CH aliph.), 1346 (C=S), 1210, 1123 (SO_2_); ^1^H NMR (DMSO-*d*_6_, δ, ppm): 3.87 (s, 3H, OCH_3_), 3.89 (s, 3H, OCH_3_), 5.94 (s, H, CH-pyrimidine), 6.54 (d, 2H, Ar–H, *J* = 9.00 Hz), 7.57 (d, 2H, Ar–H, *J* = 6.52 Hz), 8.23 (s, 2H, 2NH, D_2_O exch.), 8.68 (s, 2H, NH_2_, D_2_O exch.). ^13^C NMR (DMSO-*d*_6_, δ, ppm): 54.3 (OCH_3_), 54.9 (OCH_3_), 84.6 (CH-pyrimidine), 122.2 (2), 129.9 (2), 135.4, 141.5, 162.4 (NH–C-pyrimidine), 164.0 (C–OCH_3_), 171.0 (C–OCH_3_), 181.1 (C=S). MS (m/z): 369(M^+^). Analysis calculated for: C_13_H_15_N_5_O_4_S_2_ (369): C, 42.27; H, 4.09; N, 18.96; found: C, 41.95; H, 4.24; N, 19.18.

### **General procedure for *N-(substituted)*-4-(thiazolo[4,5-b]quinoxalin-*2(3H)*-ylideneamino)benzenesulfonamide derivatives 4 (a–j)**

The *N-(substituted)-4-thioureidobenzenesulfonamide derivatives ****2 (a–j)*** (0.01 mol) were refluxed with dichloroquinoxaline **3** (1.97 gm, 0.01 mol), in ethanol (20 ml) for 8 h. The reaction mixture was cooled, then the precipitated solid was filtered, washed with ethanol, dried and crystallized from ethanol to afford compounds **4(a-j)**, respectively.

#### **4-(Thiazolo[4,5-b]quinoxalin-*2(3H)*-ylideneamino)benzenesulfonamide (4a)**

Yield % 80; m.p.; 165–167 °C; IR (KBr,cm^-1^): 3289, 3272 (2NH), 3080 (CH arom.), 1262, 1130 (SO_2_); ^1^H NMR (DMSO-*d*_6_, δ, ppm): 2.43 (s, 2H, NH_2_, D_2_O exch.), 7.46 (d, 2H,2CH–Ar–H, *J* = 5.00 Hz), 7.48–7.68 (m, 6H, Ar–H), 7.88 (s, H, NH, D_2_O exch.). ^13^C NMR (DMSO-*d*_6_, δ, ppm): 120.4 (2), 123.3, 123.5, 126.2, 126.4 (2), 129.9, 136.2 (N=C–S), 136.3, 136.6, 143.7 (C–SO_2_), 150.3, 161.4 (C=N), 163.6 (N=C–NH). MS (m/z): 357 (M^+^). Analysis calculated for: C_15_H_11_N_5_O_2_S_2_ (357): C, 50.41; H, 3.10; N, 19.59; found: C, 50.57; H, 3.43; N, 19.41.

#### ***N-((4-(Thiazolo[4,5-b]quinoxalin-2(3H)ylideneamino)*phenyl) sulfonyl)-acetamide (4b)**

Yield % 76; m.p.; 245–247; IR (KBr,cm^-1^): 3316, 3218 (2NH), 3055 (CH arom.), 2910, 2840 (CH aliph.), 1770 (C=O), 1230, 1108 (SO_2_); ^1^H NMR (DMSO-*d*_6_, δ, ppm): 2.10 (s, 3H, CH_3_), 7.47 (d, 2H, Ar–H, *J* = 7.76 Hz), 7.59–7.88 (m, 6H, Ar–H), 8.20 (s, H, NH-SO_2_, D_2_O exch.), 8.64 (s, H, NH, D_2_O exch.). ^13^C NMR (DMSO-*d*_6_, δ, ppm): 21.2 (CH_3_), 121.3 (2), 124.2, 124.4, 124.6, 129.8 (2), 130.0, 135.5 (N=C–S), 135.8, 136.2, 136.6 (C–SO_2_), 152.1, 161.8 (C=N), 162.1 (N=C–NH), 171.3 (C=O). MS (m/z): 399 (M^+^). Analysis calculated for: C_17_H_13_N_5_O_3_S_2_ (399): C, 51.12; H, 3.28; N, 17.53; found: C, 51.31; H, 3.54; N, 17.19.

#### ***N-Carbamimidoyl*-4-(thiazolo[4,5-b]quinoxalin-*2(3H)*-ylideneamino) benzene-esulfonamide* (4c)***

Yield % 69; m.p.; > 260 °C; IR (KBr,cm^-1^): 3380–3150 (3NH, NH_2_), 3063 (CH arom.), 1220, 1111 (SO_2_); ^1^H NMR (DMSO-*d*_6_, δ, ppm): 7.44 (d, 2H, Ar–H, *J* = 4.40 Hz), 7.57–7.89 (m, 6H, Ar–H), 8.18 (s, 3H, 3NH, D_2_O exch.), 8.34 (s, 2H, NH_2_, D_2_O exch.). ^13^C NMR (DMSO-*d*_6_, δ, ppm): 123.4 (2), 124.5, 126.0, 127.9, 129.5 (2), 129.8, 136.2 (N=C–S), 137.6, 138.8, 141.1 (C–SO_2_), 151.8, 158.4 (NH=C–NH_2_), 162.2 (C=N), 163.1 (N=C–NH). MS (m/z): 399 (M^+^). Analysis calculated for: C_16_H_13_N_7_O_2_S_2_ (399): C, 48.11; H, 3.28; N, 24.55; found: C, 47.83; H, 3.59; N, 24.79.

#### ***N-(pyridin-2-yl)*-4-(thiazolo[4,5-b]quinoxalin-*2(3H)*-ylideneamino) benzene-sulfonamide* (4d)***

Yield % 72; m.p.; 196–198 °C; IR (KBr,cm^-1^): 3312, 3282 (2NH), 3078 (CH arom.), 1255, 1115 (SO_2_); ^1^H NMR (DMSO-*d*_6_, δ, ppm): 6.71 (t, H, CH-pyridine, *J* = 7.96 Hz), 6.79 (d, H, CH-pyridine, *J* = 4.08 Hz), 7.40–7.63 (m, 9H, Ar–H), 7.85 (d, H, CH-pyridine, *J* = 8.40 Hz), 8.58 (s, 2H, 2NH, D_2_O exch.). ^13^C NMR (DMSO-*d*_6_, δ, ppm): 110.1, 117.5, 121.7 (2), 124.2, 126.3, 126.4, 129.9 (2), 130.0, 135.5 (N=C–S), 136.2, 136.3 (2), 136.6 (C–SO_2_), 148.6, 151.7, 153.2 (C-pyridine), 161.4 (C = N), 164.1 (N=C–N). MS (m/z): 434 (M^+^). Analysis calculated for: C_20_H_14_N_6_O_2_S_2_ (434): C, 55.29; H, 3.25; N, 19.34; found: C, 55.18; H, 3.46; N, 19.09.

#### ***N-(Pyrimidin-2-yl)*-4-(thiazolo[4,5-b]quinoxalin-*2(3H)*-ylideneamino) benzene-sulfonamide* (4e)***

Yield % 67; m.p.; 223–225 °C; IR (KBr,cm^-1^): 3295, 3276 (2NH), 3055 (CH arom.), 1228, 1108 (SO_2_); ^1^H NMR (DMSO-*d*_6_, δ, ppm): 6.84 (t, H, CH-pyrimidine, *J* = 7.32 Hz), 7.04–7.25 (m, 8H, Ar–H), 8.64 (d, 2H, 2 CH-pyrimidine, *J* = 12.00 Hz), 8.83 (s, 2H, 2NH, D_2_O exch.). ^13^C NMR (DMSO-*d*_6_, δ, ppm): 117.5, 120.4 (2), 123.2, 126.2, 127.1, 129.9 (2), 129.9, 136.5 (N=C–S), 139.4, 140.5 (C-SO_2_), 140.8, 150.1 , 157.1 (2), 161.7 (C=N), 163.6 (N=C–NH), 168.5 (NH–C-pyrimidine). MS (m/z): 435(M^+^). Analysis calculated for: C_19_H_13_N_7_O_2_S_2_ (435): C, 52.40; H, 3.01; N, 22.51; found: C, 52.14; H, 3.32; N, 22.76.

#### ***N-(5-Methylisoxazol-3-yl)*-4-(thiazolo[4,5-b]quinoxalin-*2(3H)*-ylideneamino) benzene-sulfonamide (4f.)**

Yield % 81; m.p.; 186–188 °C; IR (KBr,cm^-1^): 3293, 3285 (2NH), 3094 (CH arom.), 2945, 2835 (CH aliph.), 1315, 1105 (SO_2_); ^1^H NMR (DMSO-*d*_6_, δ, ppm): 2.27 (s, 3H, CH_3_), 6.12 (s, H, CH-isoxazole), 7.13 (d, 2H, Ar–H, *J* = 1.36 Hz), 7.42–7.66 (m, 6H, Ar–H), 8.17 (s, 2H, 2NH, D_2_O exch.). ^13^C NMR (DMSO-*d*_6_, δ, ppm): 12.4 (CH_3_), 95.4 (CH- isoxazole), 121.7 (2), 124.4, 126.0, 127.9, 129.6 (2), 129.8, 136.1 (N=C–S), 137.3, 137.6 (C–SO_2_), 139.1, 150.4 (C- isoxazole-NH), 150.8, 161.6 (C=N), 162.3 (N=C–NH), 169.4 (CH-CH_3_). MS (m/z): 438 (M^+^). Analysis calculated for: C_19_H_14_N_6_O_3_S_2_ (438): C, 52.04; H, 3.22; N, 19.17; found: C, 51.73; H, 3.45; N, 19.36.

#### ***N-(Thiazol-2-yl)-*4-(thiazolo[4,5-b]quinoxalin-*2(3H)*-ylideneamino) benzene-sulfonamide (4g)**

Yield % 86; m.p.; 238 -240 °C; IR (KBr,cm^-1^): 3295, 3279 (2NH), 3083 (CH arom.), 1285, 1110 (SO_2_). ^1^H NMR (DMSO-*d*_6_, δ, ppm): 6.28 (d, H, S-CH-thiazole, *J* = 7.92 Hz), 6.86 (d, H, N–CH-thiazole, *J* = 7.64 Hz), 7.06 (d, 2H, Ar–H, *J* = 7.28 Hz), 7.16–7.53 (m, 6H, Ar–H), 8.07 (s, 2H, 2NH, D_2_O exch.). ^13^C NMR (DMSO-*d*_6_, δ, ppm): 112.1 (S-CH-thiazole), 121.1 (2), 123.5, 123.6, 126.3, 129.8 (2), 129.9, 136.1 (N=C–S), 136.3 (N–CH-thiazole), 136.5, 138.4 (2), 150.3, 161.9 (C=N), 162.4 (N=C–NH), 169.8 (NH-C-thiazole). MS (m/z): 440 (M^+^). Analysis calculated for: C_18_H_12_N_6_O_2_S_3_ (441): C, 49.08; H, 2.75; N, 19.08; found: C, 49.39; H, 2.48; N, 18.91.

#### ***N-(4-Methylpyrimidin-2-yl)*-4-(thiazolo[4,5-b]quinoxalin-*2(3H)*-ylideneamino)-benzenesulfonamide (4h)**

Yield % 77; m.p.; 199–201 °C; IR (KBr,cm^-1^): 3305, 3268 (2NH), 3089 (CH arom.), 2910, 2812 (CH aliph.), 1259, 1125 (SO_2_); ^1^H NMR (DMSO-*d*_6_, δ, ppm): 2.43 (s, 3H, CH_3_), 6.83 (d, H, CH-pyrimidine, *J* = 2.00 Hz), 7.46–7.67 (m, 8H, Ar–H), 7.97 (d, H, N–CH-pyrimidine, *J* = 3.28 Hz), 8.19 (s, 2H, 2NH, D_2_O exch.). ^13^C NMR (DMSO-*d*_6_, δ, ppm): 23.2 (CH_3_), 110.5, 121.4 (2), 123.7, 126.3, 126.4, 129.9 (2), 130.0, 135.5 (N=C–S), 137.3, 137.6 (C–SO_2_), 137.8, 153.20, 156.31 (CH-pyrimidine), 161.17 (C=N), 163.21 (N=C–N), 169.24 (C–CH_3_), 169.92 (N–CH-pyrimidine). MS (m/z): 449(M^+^). Analysis calculated for: C_20_H_15_N_7_O_2_S_2_ (450): C, 53.44; H, 3.36; N, 21.81; found: C, 53.31; H, 3.74; N, 22.02.

#### ***N-(4,6-Dimethylpyrimidin-2-yl)*-4-(thiazolo[4,5-b]quinoxalin-*2(3H)*-ylideneamino) benzenesulfonamide (4i)**

Yield % 75; m.p.; 246–248 °C; IR (KBr,cm^-1^): 3375, 3266 (2NH), 3077 (CH arom.), 2896, 2813 (CH aliph.), 1232, 1105 (SO_2_); ^1^H NMR (DMSO-*d*_6_, δ, ppm): 2.33 (s, 6H, 2 (CH_3_)), 6.97 (s, H, CH-pyrimidine), 7.46–7.68 (m, 8H, Ar–H), 8.14 (s, 2H, 2NH, D_2_O exch.). ^13^C NMR (DMSO-*d*_6_, δ, ppm): 24.0 (2) (2CH_3_), 110.3 (CH-pyrimidine), 119.8 (2), 123.4, 123.7, 126.3, 129.8 (2), 129.9, 136.8 (N=C–S), 137.1, 137.3 (C–SO_2_), 137.8, 150.7, 160.1 (C–N), 163.4 (N = C–NH), 167.3 (2) (2 (C–CH_3_), 169.8 (C-pyrimidine). MS (m/z): 463(M^+^). Analysis calculated for: C_21_H_17_N_7_O_2_S_2_ (464): C, 54.41; H, 3.70; N, 21.15; found: C, 54.08; H, 3.91; N, 21.44.

#### ***N-(2,6-Dimethoxypyrimidin-4-yl)*-4-(thiazolo[4,5-b]quinoxalin-*2(3H)*-ylidene-amino)-benzenesulfonamide (4j)**

Yield % 80; m.p.; 249–251 °C; IR (KBr,cm^-1^): 3296, 3230 (2NH), 3086 (CH arom.), 2916, 2839 (CH aliph.), 1222, 1117 (SO_2_); ^1^H NMR (DMSO-*d*_6_, δ, ppm): 3.75 (s, 3H, OCH_3_), 3.87 (s, 3H, OCH_3_), 5.92 (s, H, CH-pyrimidine), 7.41–7.75 (m, 8H, Ar–H), 8.24 (s, 2H, 2NH, D_2_O exch.). ^13^C NMR (DMSO-*d*_6_, δ, ppm): 54.4 (OCH_3_), 54.7 (OCH_3_), 82.41(CH-pyrimidine), 121.7 (2), 126.2, 126.3, 126.4, 129.9 (2), 130.0, 137.4 (N=C–S), 137.8, 138.1 (C–SO_2_), 138.4, 151.4, 160.8 (C=N), 162.2 (N=C–NH), 162.4 (NH–C-pyrimidine), 165.7 (CH-C–OCH_3_), 174.7 (N–C–OCH_3_). MS (m/z): 495 (M^+^). Analysis calculated for: C_21_H_17_N_7_O_4_S_2_ (496): C, 50.90; H, 3.46; N, 19.79; found: C, 50.73; H, 3.74; N, 20.12.

## Biological evaluation

### In vitro assay

#### In-vitro VEGFR-2 kinase assay

The VEGFR-2 Kinase Assay Kit [Catalog no., 40325, BPS Bioscience®, USA] is designed to measure VEGFR-2 kinase activity for screening and profiling applications using Kinase-Glo® MAX as a detection reagent. The VEGFR-2 Kinase Assay Kit comes in a convenient 96-well format, with enough purified recombinant VEGFR2 enzyme and VEGFR2 substrate. The assay protocol was carried out according to manufacturer instructions.

#### HepG2 & H9C2 cells culture and viability assay

Hepatocellular carcinoma (HepG2) and rat heart/myocardium (H9C2) were obtained from Nawah Scientific Inc., (Mokattam, Cairo, Egypt). Cells were maintained in DMEM media supplemented with 100 mg/mL of streptomycin (3810–74-0), 100 units/mL of penicillin (113–98-4) and 10% of heat-inactivated fetal bovine serum in humidified, 5% (v/v) CO_2_ atmosphere at 37 °C.

Cell viability was assessed by SRB assay ^66,67^. Aliquots of 100 μL cell suspension (5 × 10^3 cells) were in 96-well plates and incubated in complete media for 24 h. Cells were treated with another aliquot of 100 μL media containing compound 4d and Sorafenib (475,207–59-1) (Cipla Co.®, India) at various concentrations. After 72 h of drug exposure, cells were fixed by replacing media with 150 μL of 10% TCA (76–03-9) and incubated at 4 °C for 1 h. The TCA solution was removed, and the cells were washed 5 times with distilled water. Aliquots of 70 μL SRB (3520–42-1) solution (0.4% w/v) were added and incubated in a dark place at room temperature for 10 min. Plates were washed 3 times with 1% acetic acid (64–19-7) and allowed to air-dry overnight. Then, 150 μL of TRIS (77–86-1) (10 mM) was added to dissolve protein-bound SRB stain; the absorbance was measured at 540 nm using a BMG LABTECH®- FLUOstar Omegammicroplate reader (Ortenberg, Germany).

### In vivo assay

#### Animals

Male Swiss albino mice (25–35 g) were obtained from the animal breeding unit of the National Center of Radiation Research and technology (NCRRT), Cairo, Egypt.

### Ethical approval

This study was conducted in accordance with the regulations approved by the ethics committee standards and guidelines issued by the U.S. National Health Institutes in the National Research Center (NIH publication No. 4A/22) and the usage of experimental animals licensed by NCRRT animal care committee. The current study also adheres to the Arrive guidelines for reporting in-vivo experiments.

### Acute toxicity test

For the study of the compound 4d's oral toxicity, 30 male Swiss albino mice were used. They were randomly allocated into 5 groups; the first group of mice (n = 6) received a vehicle and was used as control, while the other four groups, each received a different acute dose of compound 4d dissolved in 0.05% Tween 80 (9005–65-6) (200, 500, 800, and 1000 mg/kg p.o.). Up to 14 days after treatment, clinical toxicity symptoms (death, more than 20% weight loss, diarrhea, lethargy, tremors, and convulsions) were noted ^68^.

### Irradiation

Whole body γ-irradiation of mice was performed at the NCRRT using the Gamma Cell-40 biological irradiator with a Cesium-137 irradiator unit, produced by the atomic energy of Canada limited (Sheridan science and technology park, Mississauga, Ontario, Canada). Mice were exposed to an acute dose of 8 Gy according to Yoo et al*.* ^69^.

## Experimental design

A total 32 mice were randomly allocated into 4 groups as follows: 1- control normal [Control] mice received vehicle (10% DMSO). 2- Irradiated mice (8 Gy) received vehicle [IRR]. 3- Irradiated mice treated with Sorafenib (50 mg/ kg body wt./ p.o)^70^ for 3 successive days [IRR + sorafenib]. 4- Irradiated mice treated with Compound 4d (50 mg/ kg body wt./ p.o) for 3 successive days, [IRR + cmp 4d]. After 3 days, all animals were anesthetized with ketamine (80 mg/ kg), blood samples were collected via retro-orbital plexus, and then serum samples were separated after centrifugation. Heart and liver samples were dissected and rapidly washed with cool saline then heart samples were stored at -80 °C till assay time. One lobe of liver was embedded in 10% formalin for immunohistochemistry assay.

### ELISA assay of heart creatine kinase (CK), tumor necrosis factor-alpha (TNF-α) and Caspase-9

Heart tissues of mice were weighted and minced then homogenized in cold fresh lysis buffer, the homogenate was centrifuged for 5 min at 4000 rpm, and then the supernatant was used for assay. Cardiac contents of CK, TNF-α and Caspase-9 were measured according to manufacturer instructions using CK ELISA kit [catalog no. EK730963, AFG Bioscience®, USA], TNF-α ELISA kit [catalog no. SEA626Ra, Cloud-Clone Corp®, USA] and Caspase-9 ELISA kit [catalog no. E-EL-R02054, Elabsciense®, USA], respectively.

### Assessment of serum LDH and catalase

Serum LDH was estimated kinetically using Spectrum® coloring assay Kit [Egyptian company for biotechnology, SAE®, Egypt]. It was carried according to manufacturer's instructions; the values were expressed as IU/ml.

Serum catalase was assessed colorimetrically in all groups according to manufacturer instructions using coloring endpoint kit [Biodiagnostic co.®, Egypt].

### Expression of VEGF by immunohistochemistry

Immunohistochemical study was carried out on paraffin tissue sections prepared for the detection VEGF, liver tissue sections were incubated separately with anti- VEGF (Gene Tex, USA). The expression of each marker was visualized by the chromogen 3,3-diaminobenzidine tetrahydrochloridem (DAB, Sigma Chemical Co.).

### Statistical analysis

Data were expressed as mean ± standard error (SE). Statistical analysis was carried out using one-way analysis of variance (ANOVA) test followed by Tukey- Kramer multiple comparison’s test. GraphPad Prism® software package was used to carry out all statistical tests. Figures were drawn using the Microsoft Excel program.

### Molecular modelling

The molecular docking study for compound **4d** 's structure and pazopanib was carried out using the Molecular Operating Environment (MOE) software, version 2014.090. The energy of the compound was minimised using the HamiltonianForce FieldMMFF94x. The partial charges of the forcefield were computed. The conformational stochastic of compound **4d** was analysed using the default settings. The X-ray crystal structure of VEGFR-2 in complex with sorafenib as ligand (PDB ID: 4ASD) was obtained from http://www.rscb.org/pdb. The Protein Data Bank (pdb) protein–ligand complex was prepared for docking. After threedimensional protonation of the enzyme, the system was optimised. Protein repeated chains and co-crystallized water molecules were removed. The binding pocket was determined and isolated, and the backbone was then hidden. The results were validated by redocking the sorafenib ligand. MOEDOCK was used to determine the most stable conformers' flexible docking of the ligandrigid receptor. The alpha triangle placement method and London dG as a function were used for scoring. Using the same scoring function, forcefield refinement was applied to the obtained poses. 50 of the most stable docking models of the ligand with the highest scored conformation were retained.

### Supplementary Information

Supplementary Information 1.

Supplementary Information 2.

Supplementary Information 3.

## Data Availability

The datasets generated and/or analysed during the current study are available in the supplementary files.
