# Supplementary material for: Novel VEGFR2 inhibitors with thiazoloquinoxaline scaffold targeting hepatocellular carcinoma with lower cardiotoxic impact
Source: Sci Rep. 2023 Aug 25;13:13907. doi: 10.1038/s41598-023-40832-z (PMC10457369; doi:10.1038/s41598-023-40832-z)
Supplement: Supplementary file 1 — Supplementary Information 1. [file 41598_2023_40832_MOESM1_ESM.pdf]

Biological Data availability

https://1drv.ms/u/s!Auby368jt8MLjG_RmbUExlxbMViU?e=xfGtY8
