# Supplementary figures and images for: Novel VEGFR2 inhibitors with thiazoloquinoxaline scaffold targeting hepatocellular carcinoma with lower cardiotoxic impact
Source: Sci Rep. 2023 Aug 25;13:13907. doi: 10.1038/s41598-023-40832-z (PMC10457369; doi:10.1038/s41598-023-40832-z)

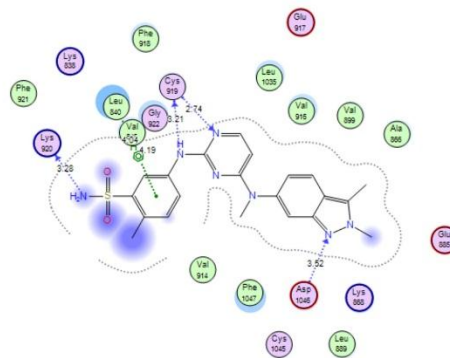

**score energy: -6.953**

Supplement: Supplementary file 2 — Supplementary Information 2. [file 41598_2023_40832_MOESM2_ESM.pdf]
