# Supplementary material for: Novel VEGFR2 inhibitors with thiazoloquinoxaline scaffold targeting hepatocellular carcinoma with lower cardiotoxic impact
Source: Sci Rep. 2023 Aug 25;13:13907. doi: 10.1038/s41598-023-40832-z (PMC10457369; doi:10.1038/s41598-023-40832-z)

^1^H NMR (DMSO-*d*_6_, δ, ppm) and ^13^C NMR (DMSO-*d*_6_, δ, ppm) for compound **4a** .


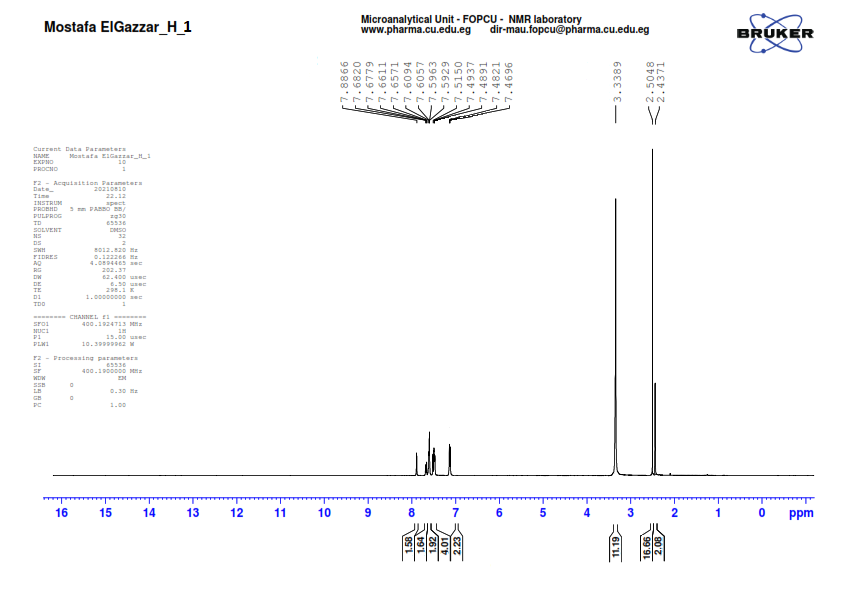


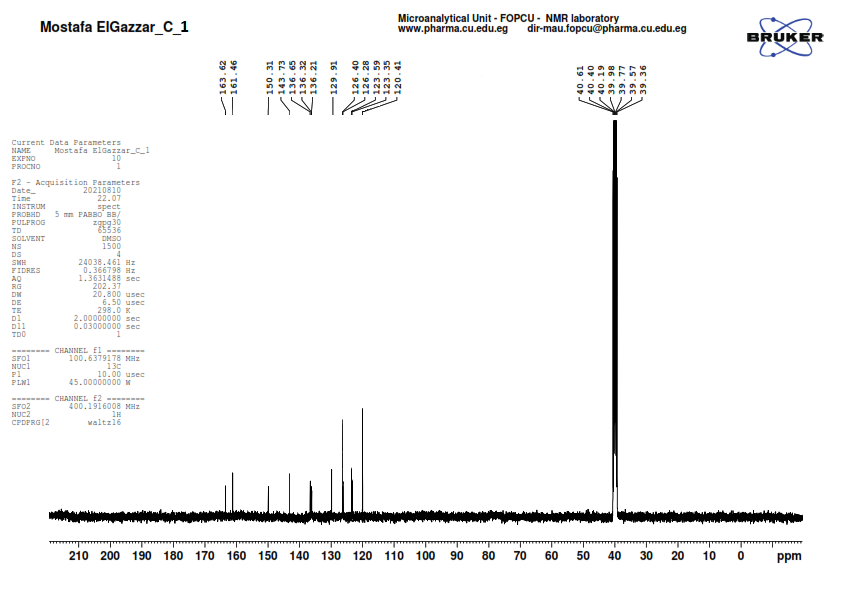


^1^H NMR (DMSO-*d*_6_, δ, ppm) and ^13^C NMR (DMSO-*d*_6_, δ, ppm) for compound **4b** .


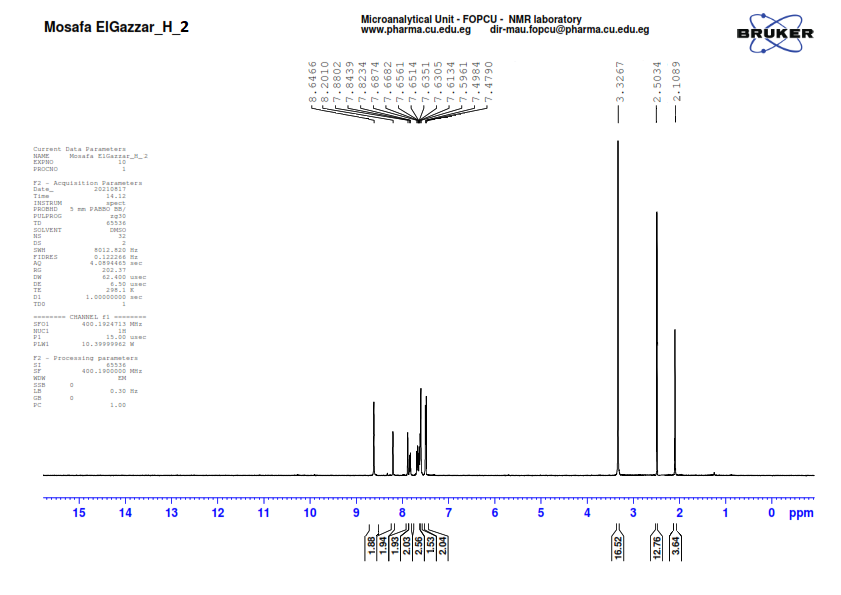


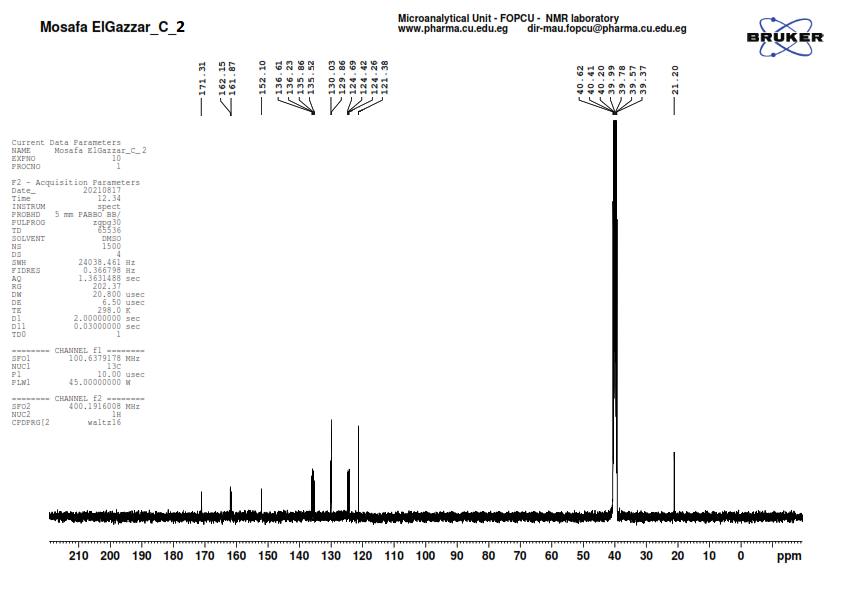


^1^H NMR (DMSO-*d*_6_, δ, ppm) and ^13^C NMR (DMSO-*d*_6_, δ, ppm) for compound **4c** .


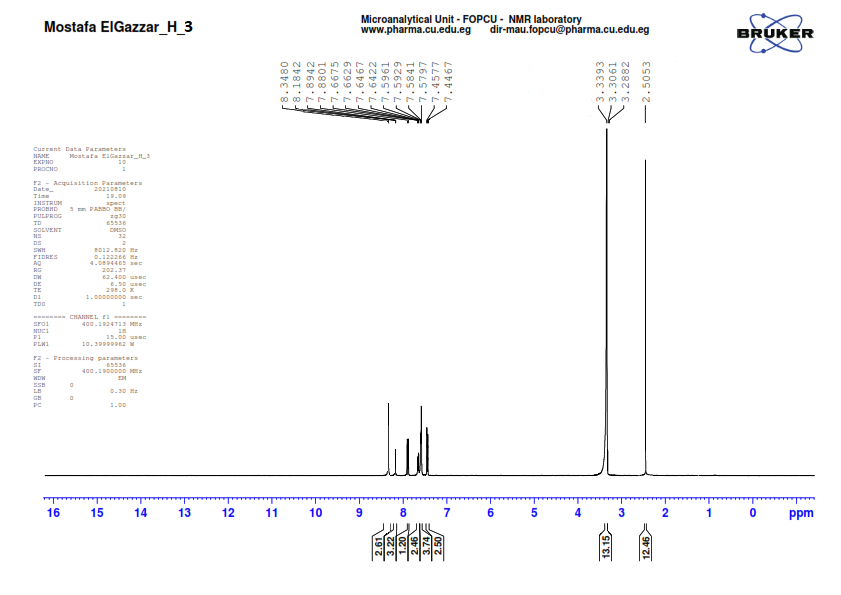


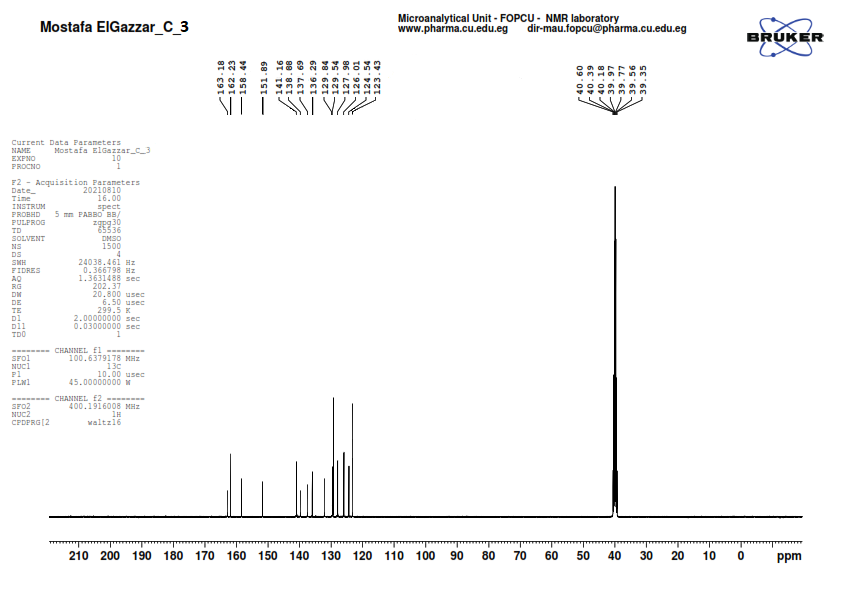


^1^H NMR (DMSO-*d*_6_, δ, ppm) and ^13^C NMR (DMSO-*d*_6_, δ, ppm) for compound **4d** .


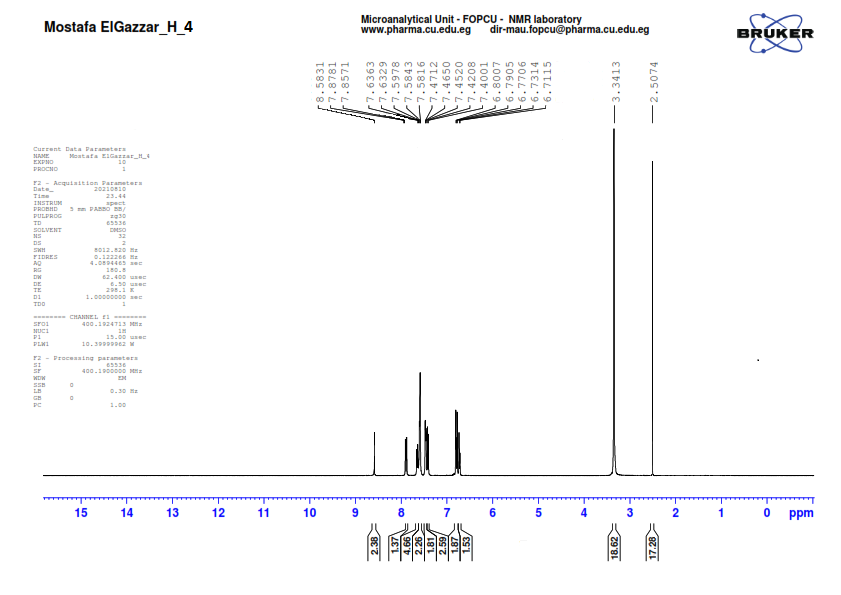


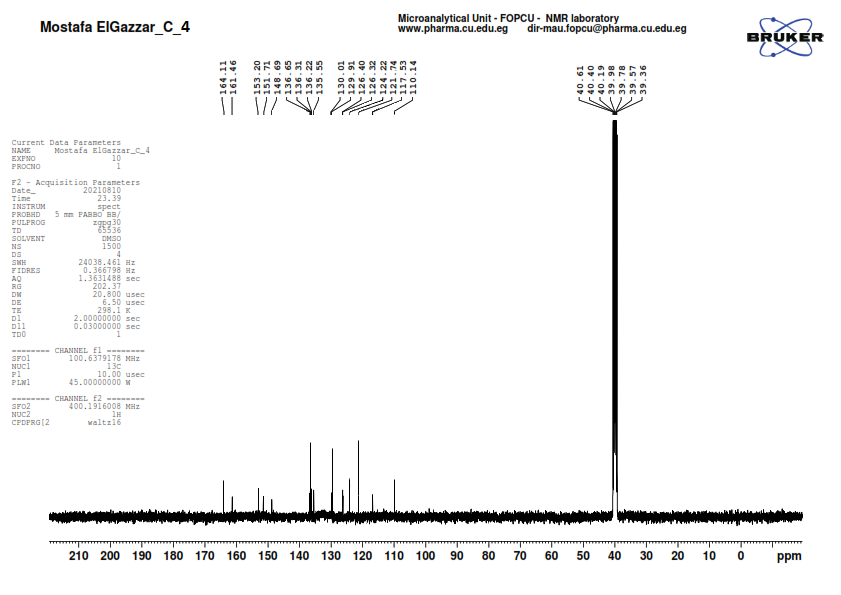


^1^H NMR (DMSO-*d*_6_, δ, ppm) and ^13^C NMR (DMSO-*d*_6_, δ, ppm) for compound **4e** .


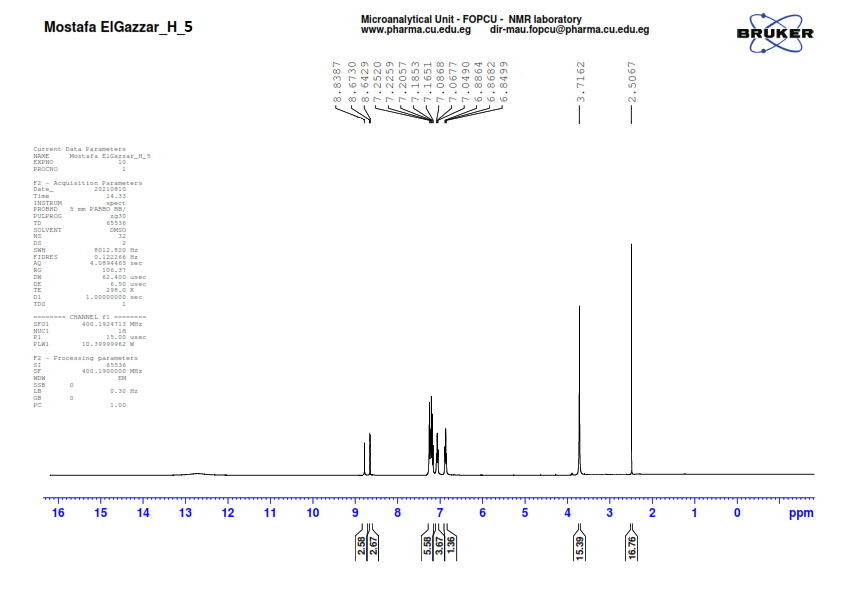


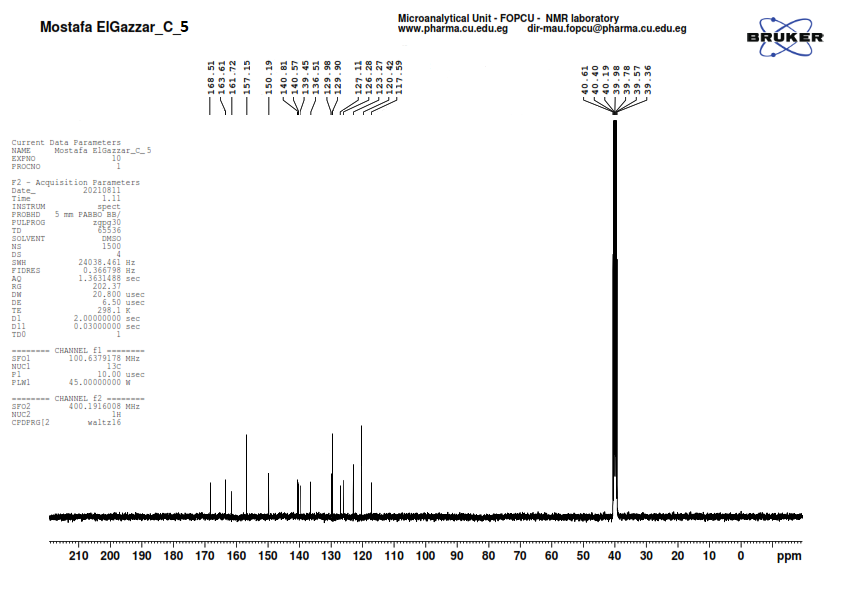


^1^H NMR (DMSO-*d*_6_, δ, ppm) and ^13^C NMR (DMSO-*d*_6_, δ, ppm) for compound **4f** .


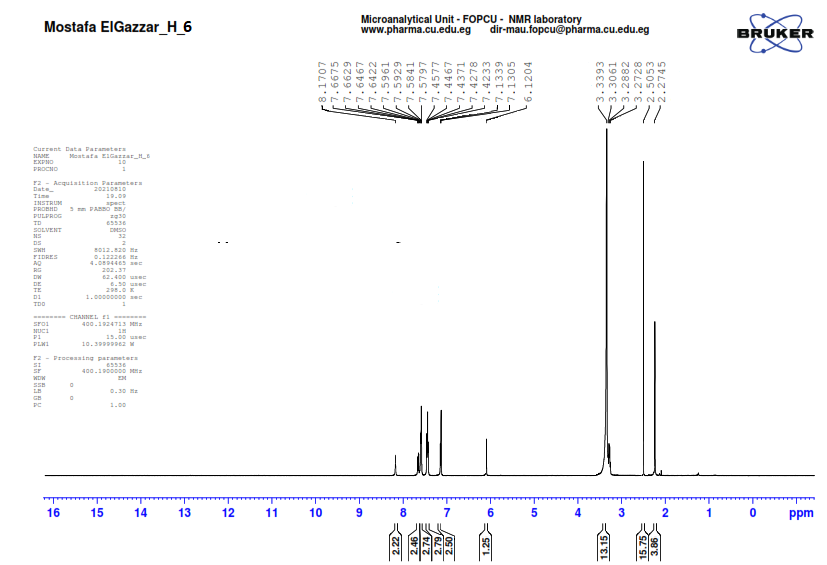


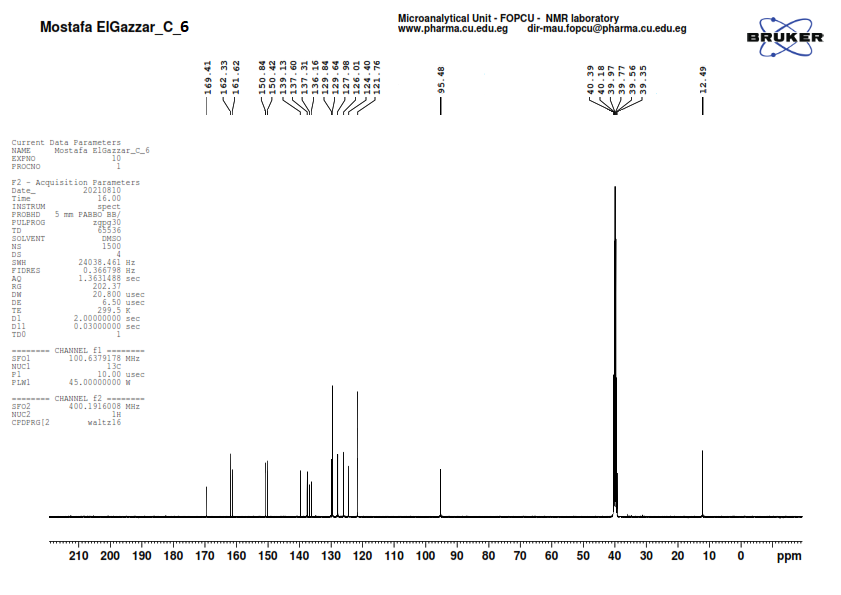


^1^H NMR (DMSO-*d*_6_, δ, ppm) and ^13^C NMR (DMSO-*d*_6_, δ, ppm) for compound **4g** .


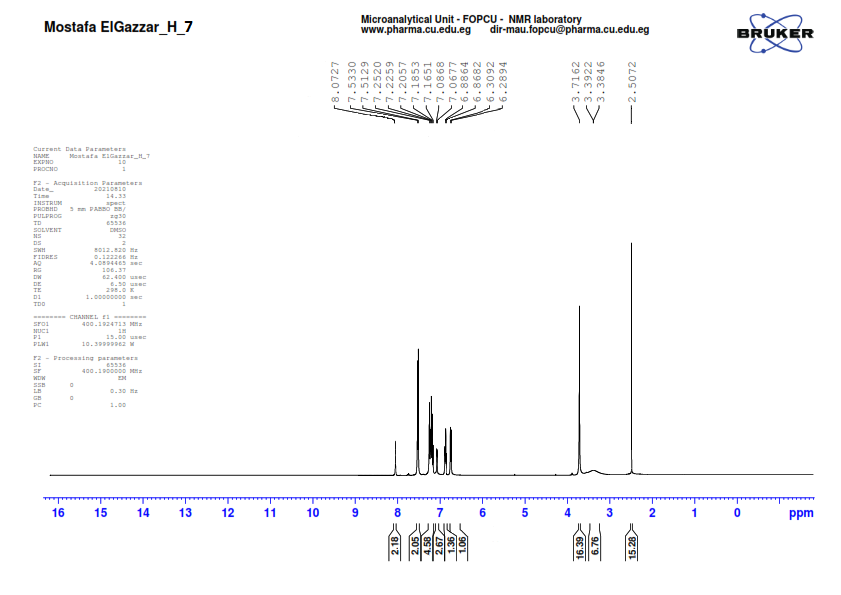


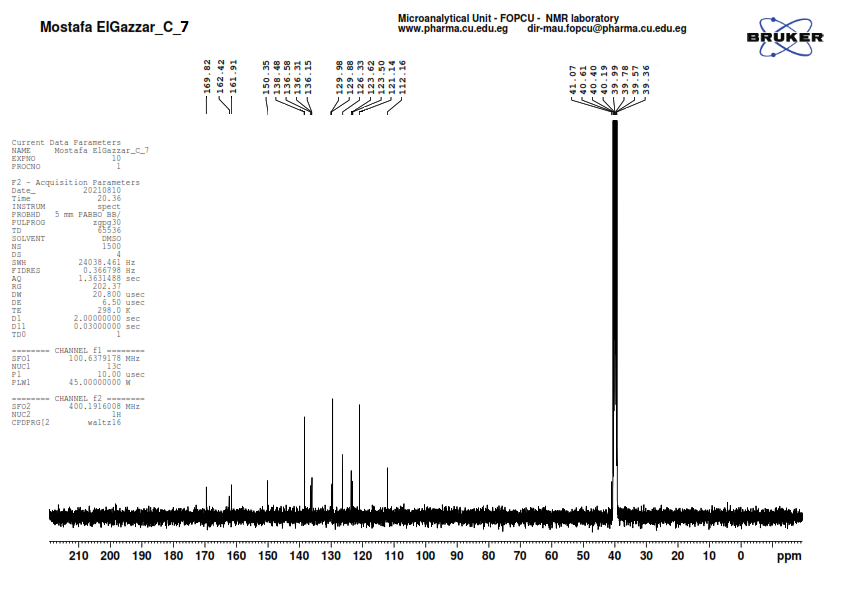


^1^H NMR (DMSO-*d*_6_, δ, ppm) and ^13^C NMR (DMSO-*d*_6_, δ, ppm) for compound **4h** .


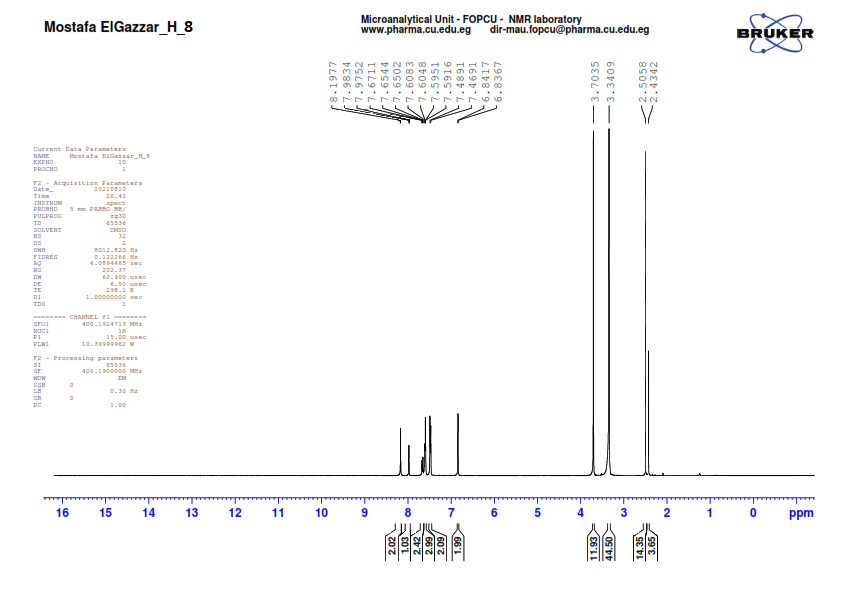


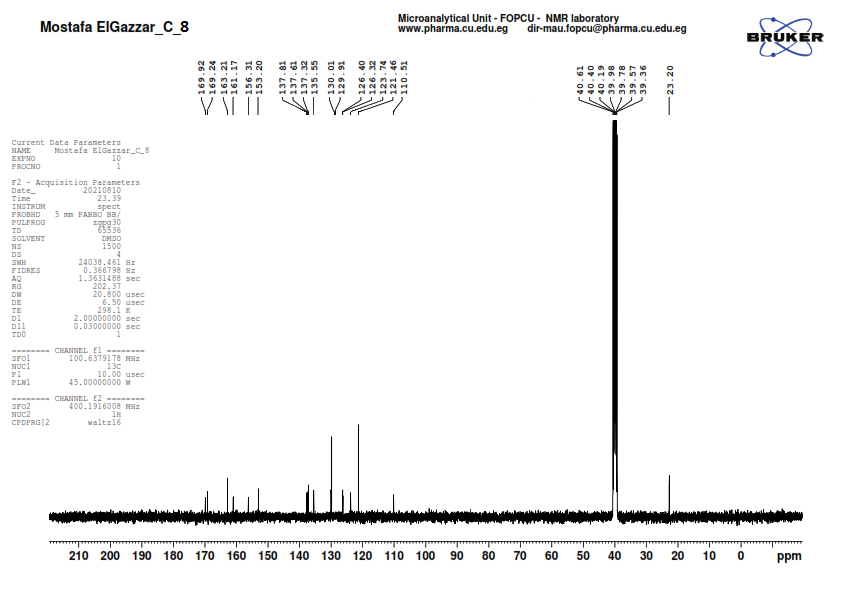


^1^H NMR (DMSO-*d*_6_, δ, ppm) and ^13^C NMR (DMSO-*d*_6_, δ, ppm) for compound **4i** .


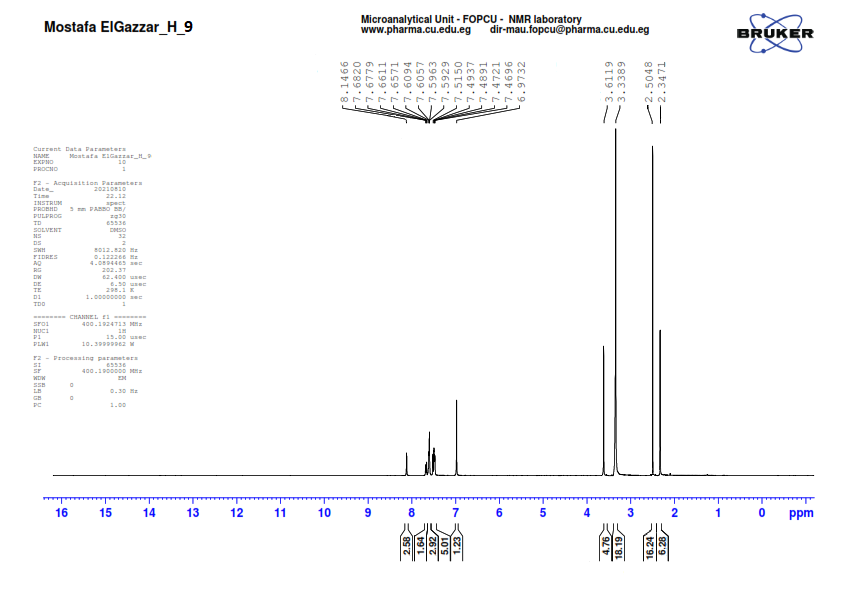


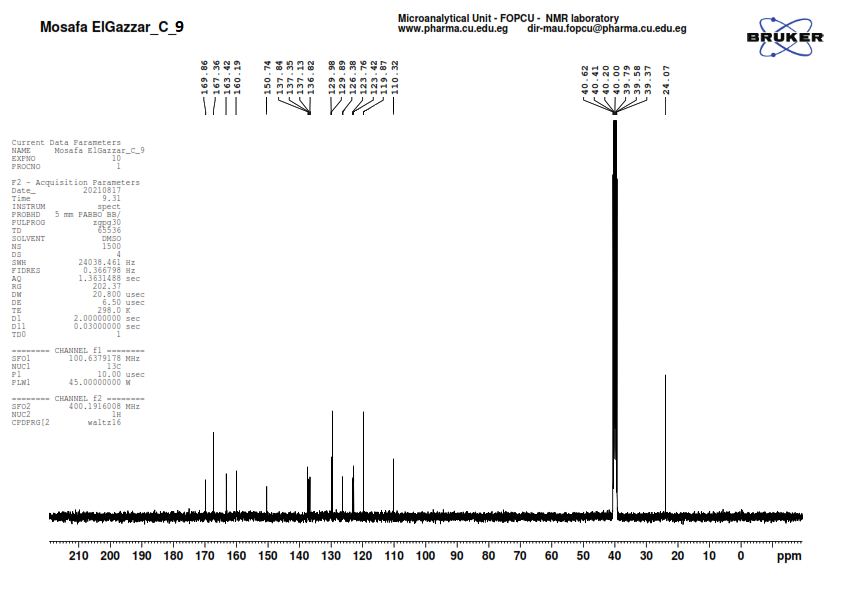


^1^H NMR (DMSO-*d*_6_, δ, ppm) and ^13^C NMR (DMSO-*d*_6_, δ, ppm) for compound **4j** .


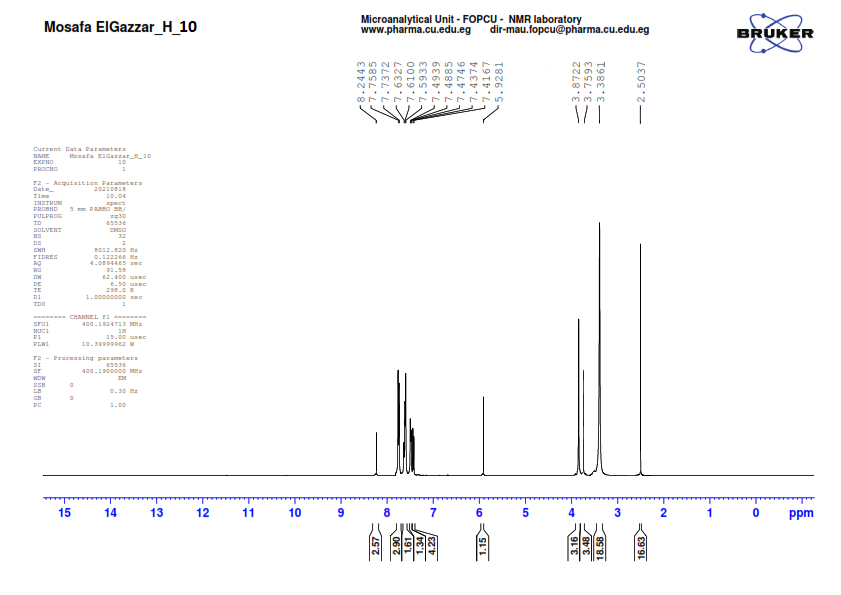


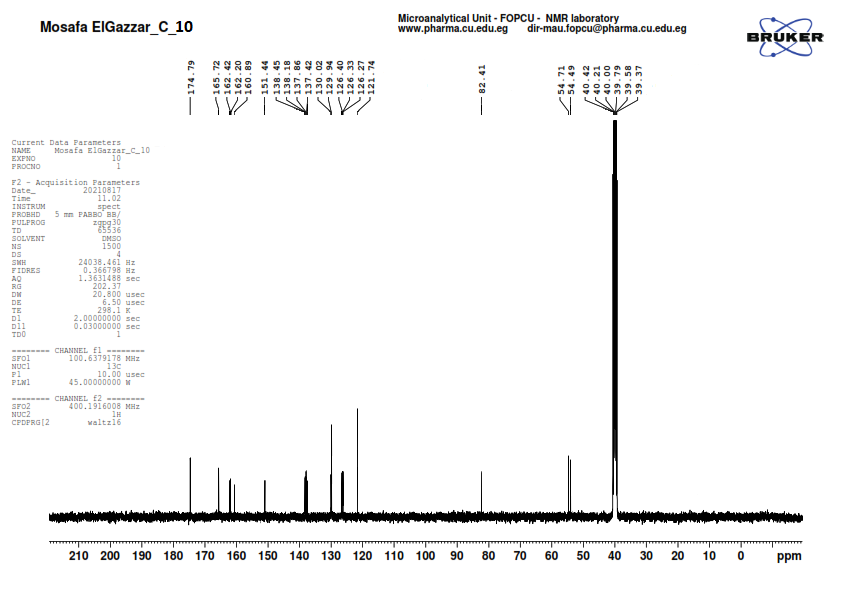

Supplement: Supplementary file 3 — Supplementary Information 3. [file 41598_2023_40832_MOESM3_ESM.docx]
